# Nano biomaterials based strategies for enhanced brain targeting in the treatment of neurodegenerative diseases: an up-to-date perspective

**DOI:** 10.1186/s12951-023-02250-1

**Published:** 2023-12-13

**Authors:** Dur E Nayab, Fakhar ud Din, Hussain Ali, Warda Arooj Kausar, Shaiza Urooj, Maryam Zafar, Ibrahim Khan, Kanwal Shabbir, Gul Majid Khan

**Affiliations:** 1 Department of Pharmacy, Faculty of Biological Sciences, Quaid-i-Azam University, Islamabad, 45320 Pakistan; 2 Nanomedicine Research Group, Department of Pharmacy, Faculty of Biological Sciences, Quaid- i-Azam University, Islamabad, 45320 Pakistan; 3 Islamia College University, Peshawar, Khyber Pakhtunkhwa Pakistan

**Keywords:** Neurodegenerative diseases, Alzheimer’s disease, Parkinson disease, Huntington’s disease, Amyotrophic lateral sclerosis, Nanomedicines, Phytoconstituents, Gene therapy

## Abstract

Neurons and their connecting axons gradually degenerate in neurodegenerative diseases (NDs), leading to dysfunctionality of the neuronal cells and eventually their death. Drug delivery for the treatment of effected nervous system is notoriously complicated because of the presence of natural barriers, i.e., the blood-brain barrier and the blood cerebrospinal fluid barrier. Palliative care is currently the standard care for many diseases. Therefore, treatment programs that target the disease’s origin rather than its symptoms are recommended. Nanotechnology-based drug delivery platforms offer an innovative way to circumvent these obstacles and deliver medications directly to the central nervous system, thereby enabling treatment of several common neurological problems, i.e., Alzheimer’s, Parkinson’s, Huntington’s, and amyotrophic lateral sclerosis. Interestingly, the combination of nanomedicine and gene therapy enables targeting of selective mutant genes responsible for the progression of NDs, which may provide a much-needed boost in the struggle against these diseases. Herein, we discussed various central nervous system delivery obstacles, followed by a detailed insight into the recently developed techniques to restore neurological function via the differentiation of neural stem cells. Moreover, a comprehensive background on the role of nanomedicine in controlling neurogenesis via differentiation of neural stem cells is explained. Additionally, numerous phytoconstituents with their neuroprotective properties and molecular targets in the identification and management of NDs are also deliberated. Furthermore, a detailed insight of the ongoing clinical trials and currently marketed products for the treatment of NDs is provided in this manuscript.

**Graphical abstract:**

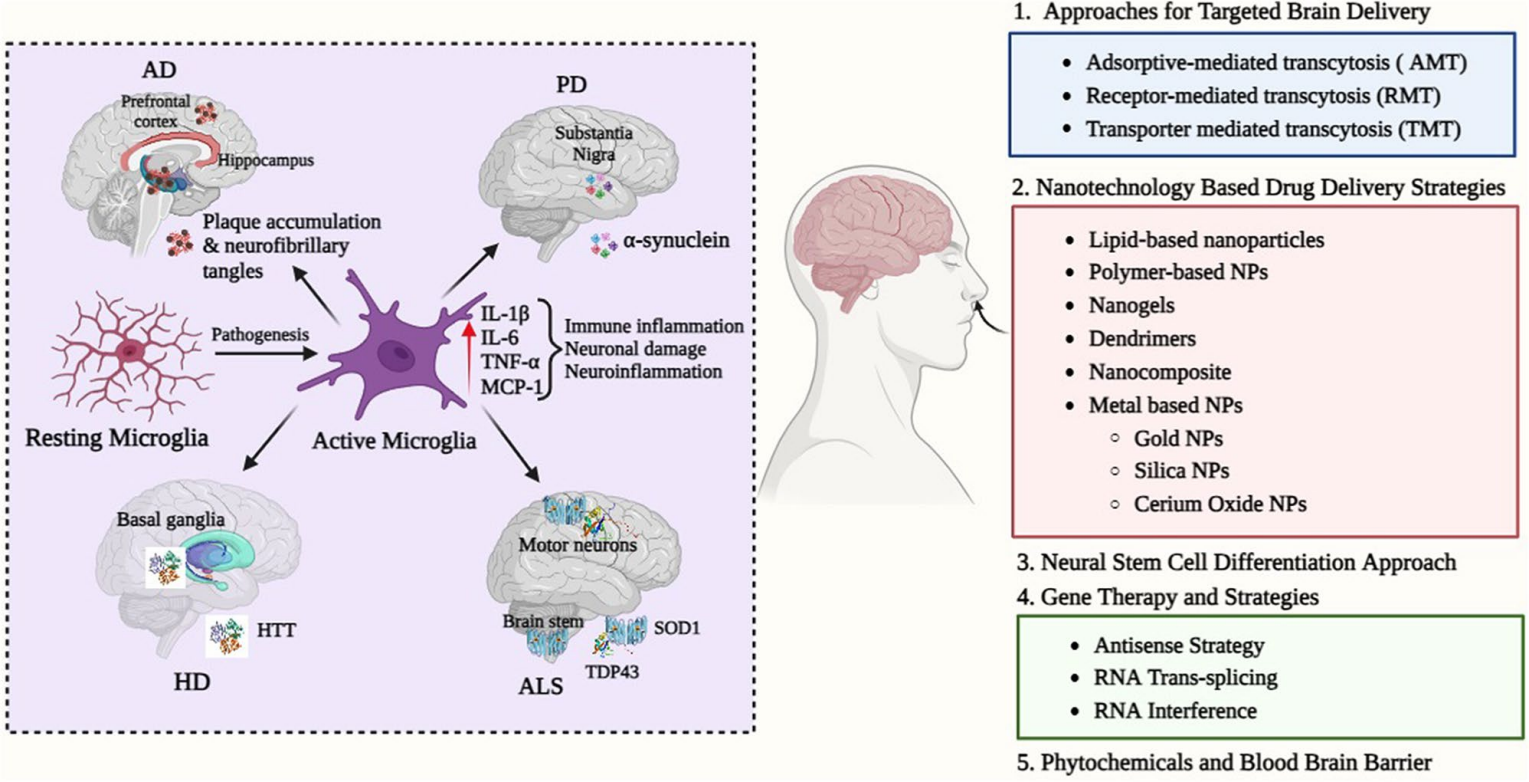

## **Background**

A distinctive feature of NDs is the progressive neuronal loss in susceptible individuals. The neuronal malfunction and their ultimate death occurred as a consequence of important physiological processes such as oxidative stress, proteotoxic stress, neuroinflammation, and apoptosis. Some people exclusively show symptoms of pure syndrome, while others shows both pure and mixed clinical symptoms [1]. It is estimated that NDs place a significant load on the healthcare system and the public due to the ensuing immunological activation of the central nervous system (CNS) [2]. Brain repair and regeneration can be aided by activating or modulating the immune system, which can help in the prevention of neurotropic viral infections and the elimination of necrotic cells [3]. However, immune activation may also result in immune-mediated disorders such as infections, immunological-mediated illnesses, and neurodegeneration. Neurodegeneration occurs slowly, but, persistently over the course of a person’s life when nerves and axons in the CNS start to die, resulting in defects in cellular function and eventually death of the cell [4]. The subsequent symptoms manifest during the degenerative phase and encompass a range of impairments, such as lack of coordination, impaired memory, and a complete inability to perform routine activities essential for maintaining a healthy lifestyle [5]. In the list of NDs, Alzheimer’s disease (AD), Parkinson’s disease (PD), Huntington’s disease (HD), and Amyotrophic Lateral Sclerosis (ALS) have been recognized as the four most common diseases. Environmental factors, impaired immunity, growing age, and the genetic makeup of the affected person are all strongly associated with these disorders [6].

Numerous layers of the neuronal sphere, spanning from the molecular to the structural regions, have shown signs of neurodegeneration. The development of effective therapeutic modalities can be aided by understanding the subcellular and molecular similarities connecting these NDs, such as abnormal synaptic behavior and the ubiquity of cerebral plaques caused by the clustering of misfolded proteins, which are related to the advancement of global research [7]. Major subcellular differences are also seen in other disorders that may affect the brain, such as cancer or stroke. It is possible for NDs to develop and progress for a variety of reasons, including DNA damage, genetic abnormalities, transglutaminase binding, protein aggregation, protein misfolding, mitochondrial dysfunction, apoptosis, autophagy, disruption of organelle membranes, and others [8, 9]. Aging is considered to be the primary contributing factor for these diseases due to the late onset of the majority of NDs. Aging can be accelerated by oxidative stress, mutations in mitochondrial DNA, and other reasons [10].

Despite the fact that more than 58 million people globally are diagnosed with AD, the frequency of cases is gradually increasing due to variables including a longer average life span as well as inherited and environmental factors. Globally, 152 million individuals are expected to be affected by the disease by 2050 [11]. In the coming years, this will significantly burden the world economy. AD is a neurological disorder that progresses irreversibly and is described by cognitive and behavioral impairment. AD results in the buildup of amyloid plaques both inside and outside of the brain’s cells, occasionally leading to cell death [12]. Although there are several conventional diagnostic techniques, they are not very sensitive to the detection of AD. Only five medications (rivastigmine, galantamine, tacrine, donepezil, and memantine) have received FDA approval for treating and reducing the severity of AD, but they are unable to cure AD, completely [13]. PD is estimated to affect one million Americans and more than ten million individuals around the globe, according to the Parkinson’s Foundation Prevalence Project, in addition to AD. Approximately USD 52 billion is expected to be borne by the US annually as a result of this disease [14]. Often called Parkinsonism, PD damages neurons that produce dopamine (neuromodulator) in the substantia nigra, a portion of the brain that produces dopamine. An imbalance between inhibitory and excitatory neurotransmitters results from this dopamine breakdown [15]. There are evidence to suggest that physical therapy, surgeries, and drugs can improve the quality of life of PD patients. Dopamine agonists, monoamine oxidase-B inhibitors, and levodopa are the main treatments for reducing motor symptoms, along with dopa decarboxylase inhibitors and, rarely, catechol-o-methyltransferase (COMT) inhibitors [16]. HD, another neurodegenerative disorder, exhibits a global prevalence of 5 to 15 cases per 100,000 individuals. This condition arises from a genetic mutation occurring in the Huntingtin gene [17]. This aberrant Huntingtin protein then fragments, causing neuronal malfunction and cytotoxicity in the affected neurons. Tetrabenazine is the most effective medication candidate against signs of cognitive impairment, followed by neuroleptics and benzodiazepines [18]. The most devastating motor neuron disease, according to the literature, is ALS, for which a remedy is yet to be discovered. The superoxide dismutase 1 gene, which has a coding sequence for the superoxide dismutase enzyme, has genetic abnormalities that account for about 20% of cases of ALS. Riluzole and Edaravone are commonly employed therapeutic interventions for ALS, despite their mechanisms of action remaining elusive [19].

**Fig. 1:**
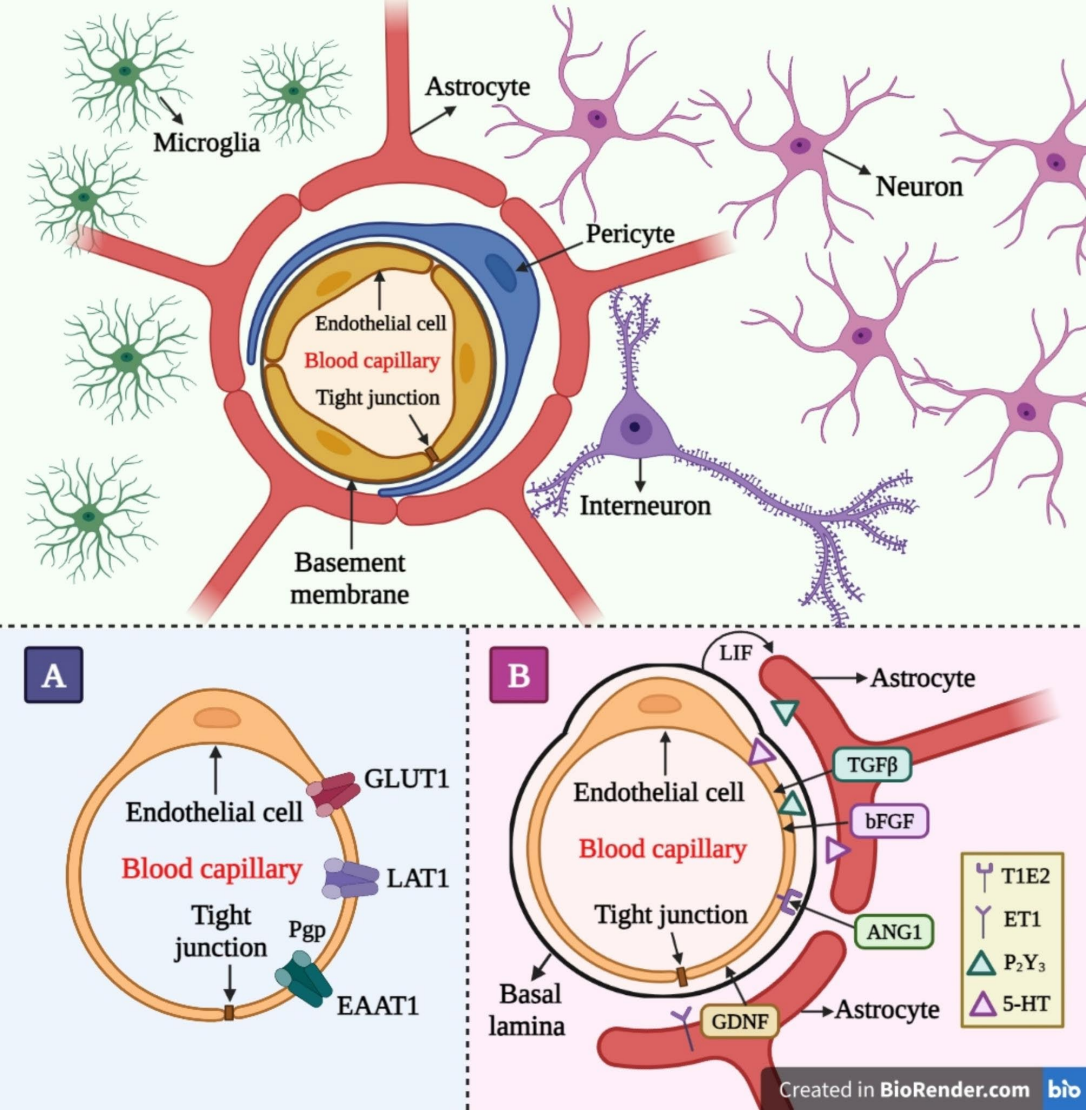
Schematic representation of the BBB, which is created by capillary endothelial cells and is bordered by basal lamina and astrocytic perivascular end-feet. Tight endothelial cell connections act as a physical barrier but also serve as a protective barrier. (**A**) The BBB serves as a robust metabolic barrier, as evidenced by the existence of various efflux transporters such as P-glycoprotein (Pgp). (**B**) Tight cellular junctions and astrocyte feet

In addition to the altered tissue homeostasis, perfusion disruption, and compromised immune systems, the pathogenesis of the aforementioned disorders starts with the accumulation and adaptation of typical host proteins. Palliative care has been used for various disorders and has thus resulted in blood-brain barrier (BBB) resistance to therapeutic drugs and all types of biomolecules [20]. The brain capillaries, which are made by brain microvessel endothelial cells (BMVECs), are responsible for these constraints of the BBB. There are some factors that contribute to the barrier function of the BBB, including tight intercellular junctions, a reduced pinocytic threshold, the articulation of efflux transporters, and the maximum level of enzymes responsible for metabolism ((Fig. 1A). The advancement of treatment methods that are tailored to specific sites and ensure safety, such as the utilization of nano biomaterials, phytoconstituents, gene therapy, and neural stem cells (NSCs), is deeply rooted in the objective of achieving the most favorable therapeutic results for these debilitating conditions [21]. The utilization of novel therapeutic nano-delivery techniques presents a potential avenue for overcoming the blood- BBB and its associated obstacles. Nanomedicine, an interdisciplinary field that combines nanotechnology with medicine, employs nanoparticles (NPs) at the nanoscale to facilitate the targeted delivery of therapeutic medicines, phytoconstituents, and biomolecules to specific anatomical sites within the body, including the brain [22, 23]. Nanotechnology is thoroughly researched with all of its known sovereignty in order to discover possible treatments for NDs [24]. NPs have made notable progress in facilitating the administration of different active molecules for the purpose of diagnosing and treating diseases. This is primarily due to their wide array of chemical properties and their capacity for chemical modification, which enables the control and enhancement of critical characteristics [25]. They possess the ability to encapsulate molecules with diverse chemical compositions, as well as provide a good delivery mechanism and protection for bioactive compounds. These carriers can improve a compound’s solubility, bio distribution, pharmacokinetics, stability, and potential toxicity [26, 27]. In addition, it has been demonstrated that NPs possess the capability to facilitate the transportation of molecules into tissues that are typically challenging to access, including the brain. Moreover, NPs offer the advantage of controlled kinetic drug release, which is crucial for sustaining long-term therapeutic interventions [28, 29]. Several types of NPs have been shown to effectively deliver drugs to neural cells in therapies related to NDs. These NPs include metal NPs, polymeric NPs, nanoparticle conjugates, solid lipid carriers, lipid/lipoprotein-based NPs, liposomes, magnetic NPs, dendrimers, inorganic NPs, nanocomposites, nanoemulsions, and antibody-tethered NPs. The utilization of these NPs aims to enhance neurogenesis and ultimately improve the efficacy of treatment for NDs [30]. In addition to that, NPs have the capability to surmount obstacles, both intracellular and extracellular, that impede the efficient transport of genes. The barriers encompass several factors such as nuclear entry, evasion of reticuloendothelial system (RES) clearance, escape from endosomes and lysosomes, safeguarding genetic cargo against degradation, release of nucleic acids, and the targeting of particular cells [31, 32]. Furthermore, the integration of gene therapy and nanomedicine has the potential to enhance the treatment of various diseases, such as monogenic disorders. Genetic modifications may be observed with the initiation of many neurodegenerative disorders. In order to formulate treatment regimens that are both successful and targeted, it is imperative to possess a comprehensive understanding of the genetic defects involved, while ensuring minimal injury to healthy cells. In due course, the efficacy of nanomedicine-based interventions in the treatment of many genetic illnesses, including cancer therapy, can be quantified [33]. The novelty of this manuscript is that it provides a more thorough analysis, focusing specifically on the advanced nanomedicine-based therapies that aims to enhance brain targeting in the context of NDs, including AD, PD, HD, and ALS. Moreover, it elucidates the obstacles pertaining to the BBB and proposes strategies for achieving targeted delivery that bypass this barrier. Furthermore, a range of nanomedicine-based therapies have been discussed, including the utilization of nanoparticle-based drug delivery systems to administer drugs, biomolecules, and phyto-constituents, the integration of nanotechnology with gene therapy to address the challenges associated with gene therapy, and the differentiation of neural stem cells (NTCs) using nanotechnology to enhance neurogenesis and facilitate the effective treatment of NDs. Additionally, a comprehensive data on marketed and ongoing clinical trials of the conventional medications and nanomedicines for the treatment of diverse NDs is appended to the tables. This range of information provided in this manuscript make it unique among the data published in the relevant field.

## Alzheimer’s disease (AD)

The neurodegenerative condition under consideration is renounced by the emergence of neurofibrillary tangles made up of tau proteins as well as the deposition of amyloid plaques [34, 35]. By 2050, the population suffering from this disease in the US is anticipated to triple. All present treatments only offer symptomatic alleviation as of right now. Five medications have been given FDA approval for use in treating AD, including galantamine, the NMDA receptor antagonist memantine, and the acetylcholinesterase inhibitors donepezil, rivastigmine, and tacrine [36]. Anti-psychotics’ efficacy as an off-label treatment has been debunked. However, curcumin, is being studied as a prospective treatment for AD because, in an animal model of the disease, it exhibits anti-inflammatory characteristics and may reduce the production of amyloid plaque [37, 38]. There are various medications used for symptom control therapies that really change how the disease develops. Additionally, more recent biological compounds have been created as possible therapies for AD. Tumor necrosis factor alpha (TNF-α) is effectively inhibited by the recombinant DNA medication etanercept, sold under the brand name Enbrel by Immunex Corporation in Thousand Oaks, California. It has been discovered that etanercept administered peri-spinally can slow the course of AD by reducing the elevated TNF-α levels linked to AD [39]. The utilization of genetic modification of nerve growth factor (NGF) was implemented in clinical studies on AD in order to impede the degeneration of cholinergic neurons [40]. A decrease in cognitive decline was observed in subjects who underwent NGF therapy. Nevertheless, the technique of intracranial administration utilizing fibroblasts encoding the patients’ NGF did not significantly decrease morbidity or mortality. Elan and Wyeth have formed a collaborative partnership to advance the development of the humanized monoclonal antibody (mAbs) bapineuzumab, alternatively referred to as AAB-001, as a potential therapeutic intervention for AD [41, 42]. As per the theoretical framework, bapineuzumab functions by binding to amyloid plaque in the CNS and subsequently removing it. Additional strategies to counteract TNF, such as the administration of low-molecular-weight TNF inhibitors or the introduction of TNF-silencing siRNA, have demonstrated considerable efficacy in mitigating the inflammatory effects associated with AD. Considerable efforts have been devoted to the treatment of AD; however, no pharmaceutical interventions have demonstrated the ability to halt the progression of this neurodegenerative disorder. At present, the efficacy of therapy often necessitates the integration of multiple treatment modalities. The optimal approach to treatment frequently involves the simultaneous management of the underlying disease as well as the accompanying symptoms.

## Parkinson’s disease (PD)

In PD, neurons that produce dopamine die off in the brain’s substantia nigra. This has been linked to the dopaminergic neurons’ buildup of ubiquitinated synuclein [34]. Parkinsonism is characterized by tremors, bradykinesia, and, in severe cases, akinesia, and it affects approximately 1% of people over the age of 60 [43, 44]. Current medications only provide symptomatic relief for Parkinson’s disease, with no approved treatment available that effectively slows down or cures the condition in its entirety. Levodopa, monoamine oxidase (MAO) inhibitors, dopamine agonists, catechol-O-methyl transferase (COMT) inhibitors, anti-cholinergic agents, and amantadine are among the therapeutic options that can be utilized. Several growth factors, such as glial cell-derived growth factor and brain-derived growth factor, have demonstrated significant benefits in terms of neuroprotection in Parkinson’s disease [44, 45]. A novel category of nucleic acid constructs has been discovered that possess the ability to effectively suppress the expression of synuclein. The potential application of RNA interference (RNAi) in the treatment of Parkinson’s disease has been explored, and experimental evidence has shown its efficacy in animal models of the disease [46]. The implementation of these novel therapies will require the development of improved delivery strategies, as they need to effectively reach the CNS to exert their intended effects. The utilization of nano-carrier devices and the intranasal administration route can effectively safeguard drugs against clearance and degradation while simultaneously facilitating the enhanced permeation of larger molecules into the CNS [47].

## Huntington’s disease (HD)

A mutant Huntingtin protein aggregates in the neurons of people with HD, which results in dysfunctional neurons and eventually neuronal death [48]. These symptoms cause the patient to move abnormally, which is known as chorea [49]. The autosomal dominant mutation is responsible for HD, a presently untreatable condition. Although there is a comprehensive understanding of the molecular and genetic aspects of HD, the current therapeutic interventions solely address the symptoms of the condition rather than targeting the underlying pathology. Patients with HD commonly experience neurological manifestations, including but not limited to depression and psychosis, in addition to the characteristic symptom of chorea. A diverse array of neuroleptic medications is being employed in the therapeutic management of chorea resulting from Huntington’s disease [50]. Standard anticonvulsants such as valproic acid are available as therapy options for some HD patients who experience seizures. HD can exhibit Parkinsonism, which is often treated with levodopa to reduce the symptoms of the condition [50]. The mutant Huntingtin protein cannot presently be treated with drugs; however, there are possible disease-modifying therapeutic ideas in research. In the HD illness model, recombinant adeno-associated viruses that can deliver RNAi therapies have been used to alleviate the disease pathology [51]. Improvement in HD has been associated with the implantation of cells that release ciliary neurotrophic factor into the brain. Innovative and potentially beneficial therapies for Parkinson’s disease include neuroprotective and disease-modifying approaches. With the use of innovative siRNA delivery techniques, it may be possible to deliver the treatments to the CNS to halt the development of HD and knock out the mutant Huntingtin protein.

## Amyotrophic lateral sclerosis (ALS)

Jean Martin Charcot initially identified ALS as a solely motor neuron illness in 1869, but it is now understood to be a multisystem neurodegenerative condition with clinical, genetic, and neuropathological heterogeneity. ALS is a neurological condition in which motor neurons are damaged. The clinical symptoms of ALS in adults include weakness of the focal muscle. The onset of weakness is mostly felt in limb muscles, firstly in the distal limb and then sometimes in the proximal limb [52]. The onset of this disease depends on age variation. There is also considerable disease variability at the genetic level, with more than 20 genes associated with ALS. The first ALS-related gene to be discovered was superoxide dismutase 1 (SOD1) in 1993; it accounts for 1–2% of sporadic ALS and 20% of familial ALS. Mutations in this gene cause SOD1 to clump, preventing it from performing its many vital physiological roles as intended. In 2008 and 2009, mutations in the genes TAR DNA-binding protein 43 (TARDBP) and fused in sarcoma (FUS) were found to produce the RNA-binding proteins TDP-43 and FUS, respectively [53, 54]. The identification of Chromosome 9 open reading frame 72 (C9orf72) in 2011 revealed its association with 30–50% of familial amyotrophic lateral sclerosis (fALS) cases and 7–10% of sporadic ALS (sALS) cases [55, 56]. Another significant factor contributing to the development of ALS is the presence of a TANK-binding kinase 1 (TBK1) mutation, leading to the manifestation of dominant autosomal ALS. ALS is assumed to be carried on by a confluence of genetic, environmental, and dysfunctional aging factors, similar to other neurodegenerative diseases [57]. So far, more than 20 genes have been linked to the illness genetically, and more genetic components are expected to be discovered. While monogenetic mutations with large effect sizes currently account for 15% of ALS cases, the disease’s genic architecture appears to be convoluted, with both common and uncommon genetic variations with small and large effect sizes increasing the risk of developing ALS.

## CNS delivery obstacles

Medication delivery to brain tissues is a particularly risky aspect of CNS treatment. It is predicted that, due to delivery difficulties, roughly 98% of microdrug molecules and 100% of proteins and nucleic acid treatment plans will be rejected from the CNS [58]. The failure in therapy is due to the innate physiochemical properties of drugs that cause failure across the blood-brain and blood-cerebrospinal fluid barriers and the dilution effects caused by systemic diffusion [59].

## The blood–brain barrier (BBB)

The BBB elucidates the underlying reasons behind the limited ability of the majority of drug molecules to permeate various tissues in body, while encountering significant challenges in traversing the brain. Paul Ehrlich is recognized as a pioneering figure in describing this phenomenon. It was observed that, with the exclusion of the brain and spinal cord, the introduction of hydrophilic dyes into the circulatory system resulted in the staining of all other tissues [60, 61]. Edwin Goldman proceeded with the investigation by administering an acid-azo dye known as trypan blue directly into the cerebrospinal fluid (CSF). The dye utilized in this experiment exhibited the ability to selectively stain the brain and CNS while not permeating the surrounding tissues [61]. The experimental results provided evidence supporting the hypothesis that there exists a physical barrier separating the circulatory system and the central nervous system. The association between the development of the mechanical barrier and the tight connections that exist between the microvasculature and the endothelial cells of the brain has been established [62]. The claudin and occludin proteins within the tight junctions establish a highly formidable barrier, effectively preventing the passage of substances between the blood and the brain [63, 64]. Upon the observation in start, it was ascertained that the BBB functions as both a chemical and metabolic barrier, in addition to its mechanical barrier properties. Brain connections are excluded by the BBB through active mechanisms such as P-glycoprotein (P-gp) and proteins associated with multi-drug resistance [65]. Furthermore, BBB cells contain a disproportionately high number of cytochrome P450 family metabolizing enzymes [66, 67]. These constraints are essential to the BBB’s functioning. Based on these characteristics, it has been hypothesized that a therapeutic moiety must be devoid of the following characteristics in order to cross the BBB: more than 10 hydrogen-bonded donors or acceptors, a molecular weight greater than 500 Daltons, similarity to the BBB enzyme system and the BBB output system, and strong binding to plasma proteins [68]. Hence, prioritizing the circumvention of the BBB should be of paramount importance in the development of CNS drug delivery systems.

## The blood cerebrospinal fluid barrier (BCSF)

The physiological surface that differentiates the CSF from the systemic circulation is commonly called the BCSF. Like the BBB, it functions to prevent the entry of toxins into the CNS. Certain molecules are allowed to traverse, functioning as a discerning barrier to generate the CSF. The choroid plexus cells are tightly interconnected, which serves as a mechanical barrier. It is noteworthy that the capillaries that provide blood supply to the choroid plexus lack tight junctions [69]. Instead, the presence of minuscule apertures within the capillaries is responsible for facilitating the supply of nutrients to this specific region. Various molecules, including pharmaceutical substances, can easily exit the capillaries through these minuscule apertures. However, the presence of epithelial tight junctions acts as a barrier, impeding their entry into the CSF. Another distinction is the larger surface area of the BCSF barrier than the BBB. The BCSF is a secondary barrier to drug distribution since its surface area is one thousand times smaller than that of the BBB [70].

## Distribution and clearance on a systematic scale

Infrequently considered obstacles to CNS drug delivery include metabolism and degradation, the dilution effects caused by systemic distribution, and drug clearance in the peripheral organs. When injected into the systemic blood circulation, these lipophilic substances more quickly pass into all other tissues in the body since the strategy of enhancing the lipophilicity of therapeutic agents has been employed to improve their permeability in the CNS [71]. With increased systemic distribution, a higher dosage is necessary to obtain therapeutic concentration in the brain. This results in systemic effects that are not targeted and increased systemic toxicity. The administration of drugs poses a significant challenge due to the limited availability of medication that can freely diffuse into the CNS as a result of its high affinity for plasma protein binding. Even though this is typically considered to be a negligible barrier to CNS administration, it is one site where innovative drug delivery technology may have a big impact. Encapsulating the medicinal substance in a carrier alters the pharmacokinetic profile overall and prevents premature release into the systemic circulation [72].

## Approaches for targeted brain delivery

**Fig. 2:**
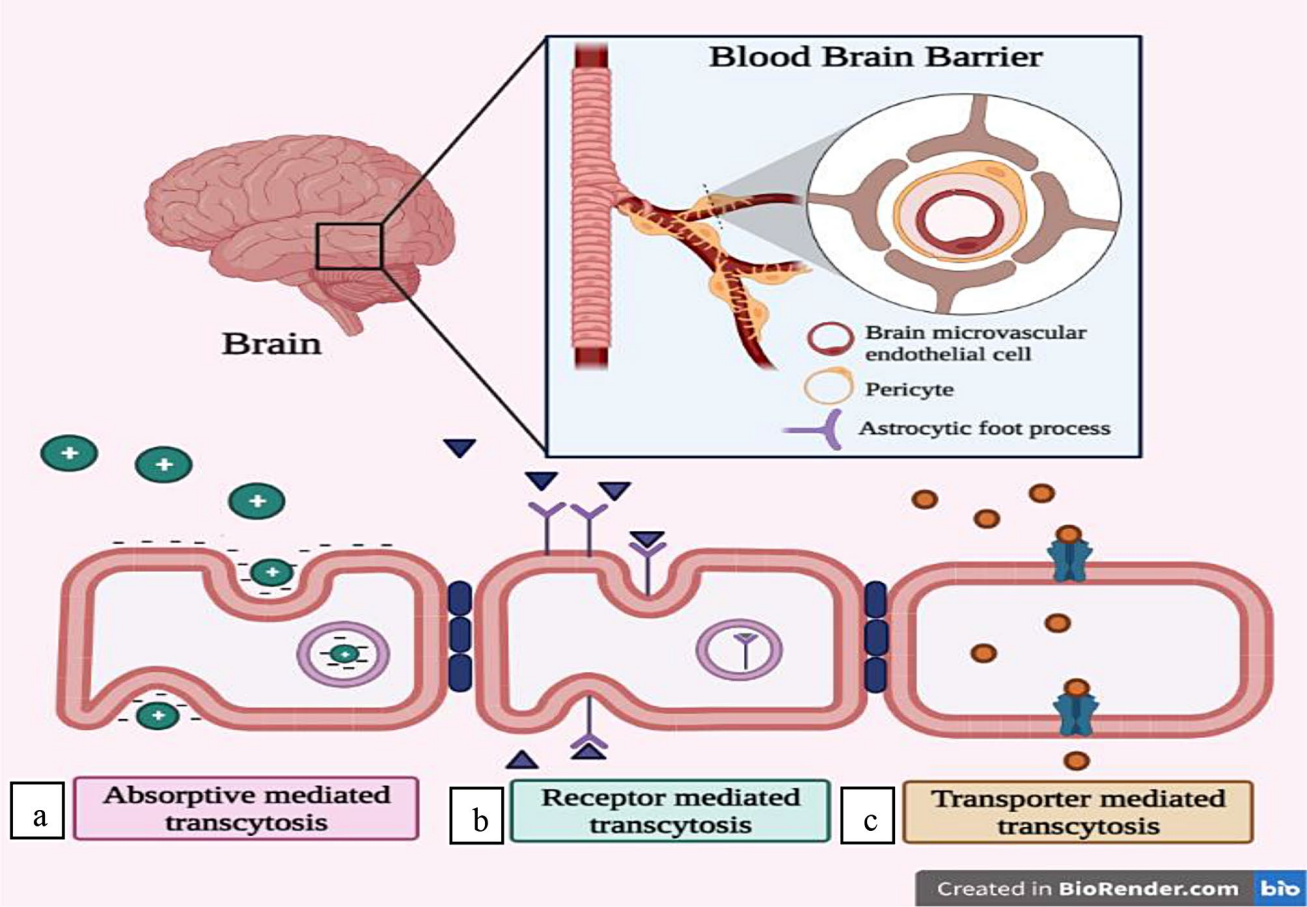
Proposed mechanisms for BBB transport. The BBB is primarily composed of a polarized layer of microvascular endothelial cells that are closely connected through tight junctions. These cells are supported by astrocytes and pericytes. Various transcellular transport processes can be identified: (**A**) Adsorptive transcytosis transports positively charged cargo like serum proteins non-specifically, (**B**) Transporting peptidic signaling and regulatory chemicals like insulin, leptin, and interleukins requires receptor-mediated transcytosis. This mechanism also helps iron and LDL transcytosis, (**C**) Transporter-mediated transcytosis is essential for transporting vital nutrients such as glucose, amino acids, and nucleosides

The gradual loss of a neuron’s structure or function, which is frequently accompanied by neuronal death, is the hallmark of NDs. The BBB, which stops the majority of medications and imaging substances from penetrating and results in adverse effects in the periphery, is one of the primary barriers [73]. Multiple endogenous transportation strategies can be used to deliver pharmaceuticals over the BBB’s tight junctions [74], as described in (Fig. 2B). Many different active targeting techniques were used to produce efficient medication delivery systems to the brain in order to address this problem. There are three main classifications of active targeting systems, namely receptor-mediated endocytosis, transporter-mediated transcytosis, and absorptive-mediated transcytosis [75].

## Adsorptive-mediated transcytosis (AMT)

There are several methods for delivering drugs to the brain that have focused mainly on a process called AMT. This method is initiated by electrostatic interaction between cationic molecules and anionic microdomains on the cytoplasmic membrane of brain capillary endothelial cells. Therapeutic drugs can be transported to the brain through AMT with the use of cationic proteins and basic oligopeptide targetors, such as cell-penetrating peptides. The utilization of cationic targetors in conjunction with AMT enables the facilitation of brain parenchyma penetration for large therapeutic molecules such as neuropeptides and proteins, as well as drug-encapsulated vectors like liposomes and NPs [76]. The BBB is suitable for the process of AMT due to its ability to facilitate the adherence of cationic molecules to the luminal surfaces of endothelial cells, followed by exocytosis at the abluminal surface. Furthermore, the BBB possesses transcytotic pathways, exhibits distinct morphological and enzymatic characteristics, and contains a significant concentration of mitochondria within cerebral endothelial cells. These features collectively facilitate the transfer of molecules across the endothelial cytoplasm [77]. During the process of AMT, a ligand engages in electrostatic interactions along the charged surface of endothelial cells. Nanomaterials that incorporate cell penetration peptides, such as transactivator of transcription (TAT)-derived peptides, and cationic proteins, such as albumin, on their surfaces have demonstrated enhanced ability to traverse the BBB [78].

## Receptor-mediated transcytosis (RMT)

The physiological mechanism that has garnered significant scholarly focus is RMT. This process leverages the abundance of receptors expressed by BBB cells to facilitate the targeted transportation of functionalized nanomedicines (NMs) across the endothelial layer of the BBB. Several proteins, such as insulin, transferrin, apolipoprotein E, and 2-macroglobulin, utilize this pathway to enter the brain and functionalize the surface of the neurovascular unit, thereby facilitating their passage across the BBB. Moreover, specific monoclonal antibodies (mAbs) that selectively bind to receptors on the BBB undergo transcytosis via receptor-mediated mechanisms [73].

**Transferrin (Tf)**: The endogenous macromolecule known as Tf is transported into the brain through the assistance of an iron ion by the transferrin receptor (TfR) located on endothelial cells. This process operates in conjunction with RMT. The liberation of transferrin Tf and iron ions from the TfR occurs through acidification within the endosomes subsequent to the process of endocytosis. Endosomes ultimately fuse with the basolateral membrane to transport their contents to the brain, bypassing the endocytic recycling pathway leading back to the apical membrane. **LDL**: There exists a transmembrane glycoprotein known as low density lipoprotein (LDL). The cellular attachment of the protein constituents of LDL particles, namely apoprotein B100 (apoB-100) or apolipoprotein E (apoE), occurs at the cell-surface region of the low density lipoprotein receptor (LDLR). Endocytosis is the process that occurs prior to this. **InsR**: According to recent research in the field of RMT, insulin receptors (InsR) expressed in endothelial cells facilitate the transportation of insulin from the bloodstream to the brain. InsR has been employed in the context of CNS drug delivery to facilitate the transportation of medication to the brain through RMT. Nevertheless, recent assertions have been made suggesting that the existence of InsR antagonists, such as S961, and the absence of InsR do not exert any influence on the absorption of insulin into the brain. **LRP1**: a constituent of the LDLR family, is involved in RMT and exhibits a diverse range of ligands, such as apolipoprotein E (apoE), amyloid precursor protein (APP), and ligand A. The expression of LRP1 is notably high in both glioma and endothelial cells. Hence, the utilization of LRP1-mediated transcytosis presents a potential strategy for targeted delivery of glioma medication to the specific affected region by traversing the BBB [79].

## Transporter mediated transcytosis (TMT)

TMT is is a promising technique for targeting the brain, as it leverages the various transport mechanisms present in the cerebral endothelium responsible for delivering vital nutrients and endogenous substances to the brain. TMT is a process that selectively takes up and delivers medications into the brain, as it only allows the passage of drugs that closely resemble the endogenous substrates. There are numerous potential applications for glucose transporters (GLUT) in the context of brain targeting, as they facilitate the transportation of glucose from the bloodstream to the brain. In the mouse brain, The GLUT1 glucose transporter facilitates the translocation of liposomes containing mannose through the BBB. Another notable mechanism for transport is through the choline transporter, which has the ability to selectively bind to positively charged quaternary ammonium groups or simple cations [75].

## Nanotechnology-based drug delivery strategies

**Fig. 3:**
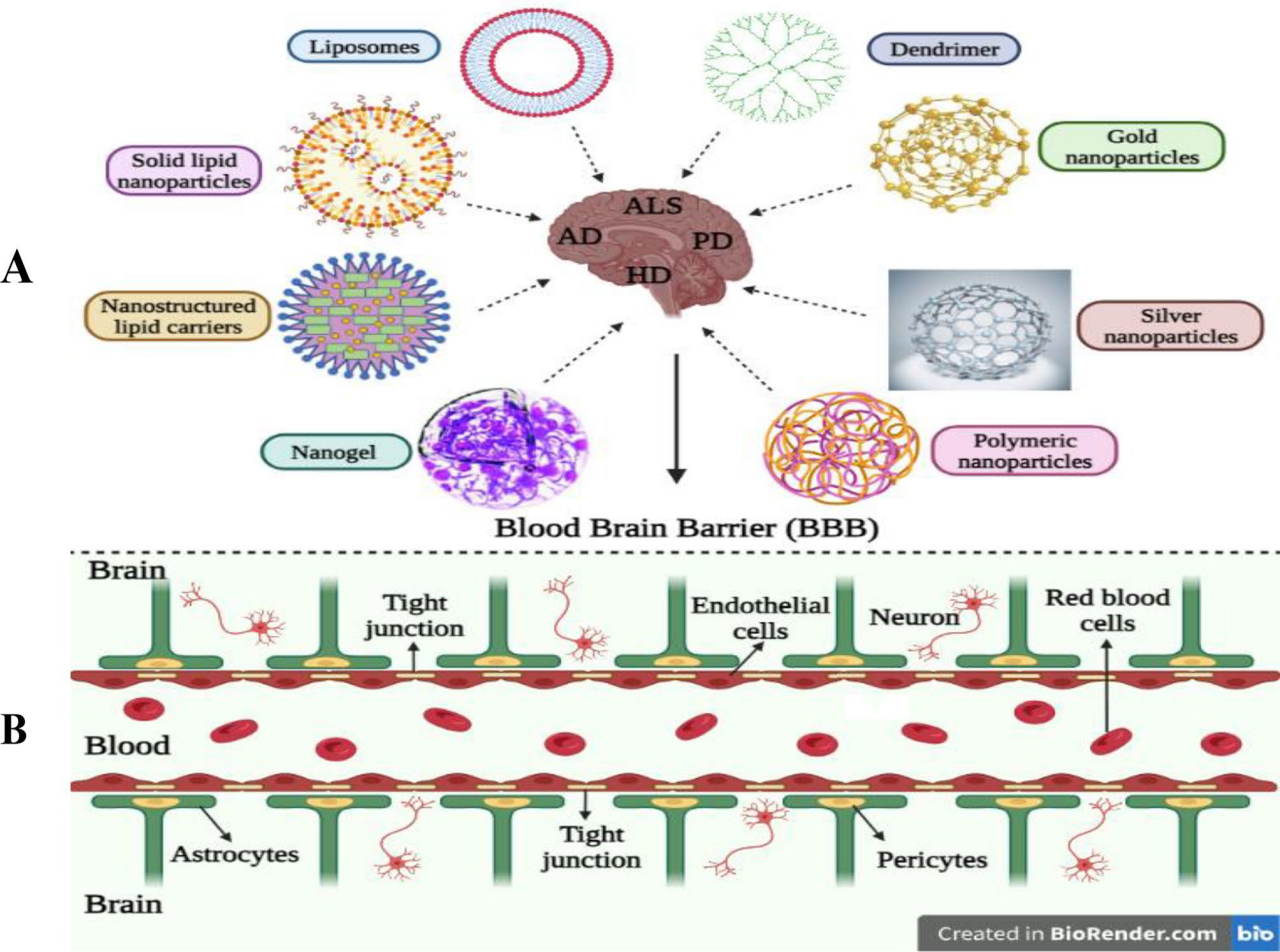
Illustration of various delivery systems based on nanotechnology including, liposomes, solid lipid nanoparticles, nanostructured lipid carriers, nanogels, polymeric nanoparticles, silver nanoparticles, gold nanoparticles and dendrimers for the treatment of neurodegenerative diseases. **B**) Represents the mechanisms of passages of the nanoparticles through BBB.

Various nanotechnology-based drug delivery strategies are discussed below to explore their ability to cross the BBB and achieve desired therapeutic effects. Illustrative images of the nanoparticles along with their representative mechanisms of passages through BBB is given in (Fig. 3C).

### Lipidbased nanoparticles (LNPs)

LNPs have emerged as a prominent technology for the controlled delivery of drugs to the brain. These systems present a compelling choice for drug nanocarriers owing to their inherent safety, biocompatibility, and biodegradability. The inherent lipidic composition of these entities is analogous to the lipidic composition of the BBB, thereby facilitating their transcellular passage into the brain. The central composition of LPNs often comprises phospholipids, fatty acids, monoglycerides, triglycerides, fatty alcohols, and waxes [80, 81]. Liposomes, solid lipid nanoparticles (SLNs), and nanostructured lipid carriers (NLCs) are widely recognized as the three primary classifications of LNPs that are frequently encountered in both research and practical applications, particularly in the context of CNS diseases. These LNPs have garnered significant attention due to their potential for effectively managing such diseases [82, 83]. The three types of LNPs exhibit variations primarily in terms of their lipid layer organization, shape, loading capacity, average particle size, and electrokinetic activity. This particular nanocarrier demonstrates enhanced versatility through its capability to encapsulate drugs with both hydrophilic and hydrophobic properties [84, 85]. Nevertheless, there are certain limitations that hinder their mass production and consequently impede their widespread utilization [86, 87]. Some of these drawbacks include low stability, a complicated production process, unexpected polymorphism transitions, and drug release during storage. Notwithstanding these constraints, LNPs such as SLNs and NLCs are swiftly emerging as promising diagnostic [88] and therapeutic modalities for AD and other CNS disorders, such as anxiety and depression [89, 90]. Recent preclinical studies have paired modern LNPs with AD animal models, with promising outcomes. Almuhayawi et al. developed pomegranate extract-loaded LNPs and tested them in an aluminium chloride-induced rat model of Alzheimer’s disease. Pomegranate extract contains rich antioxidants, alkaloids, and tannins. These LNPs improved cognition ratings, brain homogenate antioxidant biomarkers, NFTs, and Aβ deposits compared to untreated controls [91].

Curcumin-loaded LNPs were studied for their potential neuroprotective benefits in an AD mice model by Giacomeli et al. Curcumin’s pharmacological effects are constrained by its low solubility and, by extension, its low bioavailability, even though it has been shown to have therapeutic potential for a variety of ailments. In order to improve solubility, the authors of this work created a hybrid nanocarrier composed of a lipidic core and a PLC shell. Serum levels of biomarkers for neuroinflammation in the hippocampus and cortical homogenates were decreased in animals fed this nanocarrier, and the animals’ spatial memory was enhanced [92]. Pinheiro et al. mixed natural ingredients with LNPs. Quercetin, a flavonoid found in a wide variety of fruits and vegetables, served as the load’s bioactive component. It’s an excellent source of antioxidants. To improve LNP transport across the BBB, the researchers coated the LNPs with Tf. These LNPs were reported to increase quercetin’s penetration over the BBB without causing toxicity to hCMEC/D3 cells, a popular in vitro model of the human BBB [93]. In a separate investigation, it was observed that the utilization of curcumin loaded NLCs resulted in enhanced tissue architecture and a reduction in the expression of p-NF-κB, TNF-α, and COX-2 within brain tissues. These findings indicated a potential neuroprotective effect of curcumin NLCs in the context of depression and anxiety [94]. The sesquiterpene alcohol bisabolol has a weak solubility and hence a limited bioavailability, despite having anti-microbial, anti-inflammatory, anti-cancer, and anti-cholinesterase activities. Sathya et al. used the rapidly growing mouse neuroblastoma cell line Neuro-2a to create bisabolol-loaded cholesterol LNPs, which they then tested for their neuroprotective effects in an amyloid beta (Aβ) generated in vitro model. These data demonstrate that these LNPs can inhibit Aβ aggregation, upregulate Bcl-2 proteins, and downregulate Bax expression, all of which serve as antioxidants and protect Neuro2a cells from Aβ-induced neurotoxicity [95]. Recent research by Dara et al. examined the therapeutic potential of erythropoietin (EPO) in AD. EPO is neuroprotective in a variety of circumstances, including diabetic neuropathy, cerebral ischemia, epilepsy, and spinal cord injury [96]. In PD and AD, EPO modulates neurogenesis while simultaneously promoting neuronal survival. Due to its hydrophilicity, rapid circulation clearance, and large molecular weight, EPO can only penetrate a limited portion of the BBB. When EPO was encased in SLNs and administered to a Westar rat model of AD, it significantly decreased oxidative stress and Aβ formation in the hippocampus. In addition, rats treated with EPO-LNP had enhanced spatial memory compared to their non-treated littermates [97].

### Polymer-based NPs

Polymeric NPs are widely employed for the delivery of a variety of drugs because of their long shelf life, improved biocompatibility, and biodegradability, all of which allow for regulated drug release at particular sites [98]. Synthetic polymers, including poly lactic acid, poly glycolic acid, and polyacrylates, are often used in the manufacture of NPs, along with natural polymers like chitosan, alginate, and albumin. The aim of using biodegradable PLGA NPs to treat AD is due to their BBB permeability and neuroprotective potential [99]. Curcumin, a polyphenolic compound with antioxidant and anti-amyloid properties, is an effective therapy for AD. However, its utility is severely constrained by its poor water solubility; this problem can be addressed by fabricating NPs [100]. Mathew et al. created Tet-1 peptide-linked curcumin as a result. Its application is severely constrained by its poor water solubility; however, this problem can be addressed by PLGA-loaded NPs and studies into the targetability of the brain for AD. Results showed that NPs were drawn to neurons and that Tet-1 aided in BBB translocation. The produced NPs are anti-amyloid and have anti-oxidative properties without being damaging to cells [101]. Curcumin’s inherent properties are mostly unaffected by the PLGA encapsulation process. Curcumin-loaded PLGA NPs were intravenously administered to the adult rat hippocampus and subventricular zone to stimulate neurogenesis in a separate investigation [102]. The generated NPs induced in vitro cell proliferation and brain development-related gene expression by activating the Wnt/catenin pathway. NPs can trigger neurogenesis and remediate learning and memory problems in amyloid beta-producing rats. PLGA curcumin NPs may improve the brain’s ability to mend itself, making them a promising treatment option for AD [103]. Diterpene andrographolide (AG) can reduce inflammation caused by NDs. However, AG’s limited usefulness is due to its low bioavailability. NPs based on poly ethyl cyanoacrylate (PECA) and human serum albumin (HSA) were created to circumvent the limitations of AG in human serum. The emulsion-polymerization method was used to create PECA NPs, whereas the chemical cross-linking method was used to create HSA NPs. The ability of AG-NPs to traverse the BBB was evaluated using an in vitro model based on the human cerebral microvascular endothelial cell line (hCMEC/D3). Compared to HAS NPs, which increased AG permeability while keeping the BBB intact, PECA NPs, which quickly damaged the cell layer, had the opposite effect. HSA can, thus, be considered a plausible BBB-penetrant andrographolide trafficking candidate [104].

NPs of PLGA-functionalized quercetin were tested for their ability to inhibit and disassemble Aβ-42 fibrils. Synthesized NPs improve neuron cell survival and mitigate Zn2+/ Aβ42-induced neurotoxicity. Moreover, behavioral investigations reveal that PLGA-QT NPs contribute to the rehabilitation of memory and cognition deficits in APP/PS1 mice [105]. In a distinct experiment, PLGA NPs with phytol encapsulation were produced via solvent evaporation. The form, in vitro drug release, AchE inhibitory activity, antiaggregation and disaggregation, antioxidant activity, and cytotoxicity of the NPs were all evaluated. According to the findings, NPs had a spherical shape, a smooth surface, and a nanoscale dimension. In Neuro2a cells, phytol-PLGA NPs destroyed amyloid aggregates and decreased AchE activity while showing modest cytotoxicity [106]. According to the aforementioned study, phytochemical-encapsulated nanocarrier systems that can cross the BBB offer new hope and a novel treatment strategy for AD.

### Nanogels

Nanogels have gained recognition as a promising targeted drug delivery platform depending on their notable attributes, including elevated colloidal stability, substantial drug loading capacity, core-shell architecture, exceptional permeation characteristics, and responsiveness to environmental stimuli [107, 108]. The penetration capabilities of these nanoscale drug carriers in the brain parenchyma are superior to those of conventional nanomaterials, as demonstrated in both in vitro and in vivo studies [109]. Given its capacity to be nanoengineered and bypass physiological barriers, nanogel-based technology is highly suitable for the treatment of neurological ailments, i.e., neurodegenerative disorders, through non-invasive means. The term “NanoGel” was initially introduced by Vinogradov et al. in 1999 to refer to hydrophilic polymer network particles. These particles have the ability to transport antisense oligonucleotides and are synthesized through the crosslinking of polyethylene glycol with polyethyleneimine (PEG-PEI) [110]. Three-dimensional colloidal hydrogel NPs have a diameter of less than 200 nm and combine hydrogel and nanocarrier system benefits [111]. Nanogels have been linked to each other through physical or chemical covalent bonding for production purposes. Nanogel network structures include hollow, hairy, multilayered, core-shell, and core-shell core cross-linking [112]. They can also be categorized by how they react to outside cues [113]. Overall, nanogel technology has the potential to provide cutting-edge treatment for CNS disorders. Consequently, the design of cost-effective, safe, and more potent nanomedicines has become a significant focus of numerous nanoformulation studies. Various nanogel-based approaches are now feasible due to the ongoing formulation of novel nanogel technologies, such as surface modification of receptors, ligands, and magnetic structures, which can be used to circumvent the BBB and deliver targeted medications. Nanogel-based technologies are being utilized in the creation of nanovehicles for different neuroprotective drugs, for example, nucleic acids, peptides, nerve growth factors, and free radical scavengers. The current state of nanogel drug delivery methods is characterized by their early developmental stages [114]. Therefore, before these treatments can be widely used in the clinic to address several ailments, however, deep research on nanogel systems is required, including a variety of preclinical and clinical trial investigations. Engineered PEGylated nanogels were loaded with dopamine and modified transferrin receptor ligands. Animal models revealed that the level of dopamine in the brain loaded through the nanogel obtained was nine times greater than the free dopamine administered. The excision of Lewy bodies is another effective PD treatment strategy [115]. Curcumin and epigallocatechin-3-gallate (EGCG) are two powerful compounds that can prevent amyloid aggregation. A hyaluronic acid (HA) alteration served as the connecting link between these two inhibitors. The results demonstrated that, as compared to EGCG- or EHA-loaded single-modified nanogels, the dual-modified nanogels (CEHA) produced 69 and 55% greater inhibition. The laboratory study revealed that CEHA dramatically increased SH-SY5Y cell survival [116].

### Dendrimers

The smallest, most precisely specified nanoformulations, dendrimers, are used in biomedicine to treat NDs. The size, core-shell composition, and surface functional groups of dendrimers can be modified to generate a diverse range of nanocarriers that facilitate the delivery of medications and genes to the brain [117]. Importantly, dendrimers’ robust antiamyloidogenic action opens up a wide range of therapeutic possibilities for the diagnosis and treatment of prion disorders, AD, and PD [118]. Interesting anti-amyloidogenic tools have also been developed using PAMAM dendrimers. Amyloid deposition of the 28-amino-acid Aβ peptide (Aβ 1–28) was prevented, and existing fibrils in amorphous forms were disaggregated by three generations of PAMAM dendrimers (G3, G4, and G5). The effect was proportionate to the number of dendrimers produced, and its intensity rose as the concentration did. The amyloid aggregation process was most effectively disrupted by PAMAM dendrimer generations that were bigger in size [119]. In contrast, a rotenone-induced mouse model of PD has described cationic carbosilane (CBS) dendrimers as effective therapeutic agents. Dendrimers exhibited a dual neuroprotective action because of their ability to both encapsulate and complex rotenone and inhibit fibrillation of α-syn. Intriguingly, this type of dendrimer was superior to PAMAM, phosphorous, and viologen-phosphorous dendrimers in protecting against rotenone damage [120]. Phosphorus-containing dendrimers are also used to sterilize medical equipment to prevent the spread of prion infections. Dendrimers show promise as a vehicle for systemic delivery of therapeutic medicines for the treatment of AD. Its pricey production is just one of several factors that limit its usefulness. Long-term dendrimer exposure and its potential health implications must also be studied [121]. Similar in safety to linear PEG or dextran, dendritic polyglycerol derivatives (dPG) are another type of dendrimers that can be used for systemic drug delivery. Although further biocompatibility studies in more complicated biological models are needed, dPG microspheres loaded with dimethilfumarate and curcumin have the potential to treat MS and have shown a good long-term release of both drugs with low in vitro toxicity [122].

### Nanocomposites

A composite is a substance that combines the properties of multiple different materials. A matrix and maybe a reinforcing phase make up this structure. One-dimensional objects include tubes and threads; two-dimensional objects include layered materials like clay, and three-dimensional objects include spheres [123]. In general, the following characteristics are used to categorize composites: Particulate composites, which are made up of particles embedded in a matrix; laminated composites, which are made up of thin layers of fully bonded materials; and fibrous composites, which are made up of fibers intertwined in a matrix. These are the three main categories of composite materials. These composites are called “polymer-based composites” when a polymer is used as the matrix. The importance of using polymers or polymer-based composites to generate multifunctional materials has been rightly emphasized in the literature for biomedical applications. Recent research in laboratory and clinical settings has focused on a wide range of biodegradable and bioresorbable materials and design advances for biomedical devices [124]. Load-bearing applications, tissue engineering, and drug delivery are just a few of the many fields where polymer-based composites have shown promise. It is possible to tailor a composite material’s chemical, physical, and mechanical properties; in fact, the end product may exhibit intriguing features that the individual constituents often lack while combining the greatest traits of its constituents. In polymer-based composites, the interaction between the matrix and the reinforcing phase, which can be either continuous or discontinuous fibers or micro/nanoparticles, is a major contributor to the composite’s performance [125]. As our knowledge of the pathogenic processes underlying AD/PD has grown, new molecular targets that may mitigate neurodegeneration have been identified. For the treatment of cognitive deficits and the avoidance of oxidative stress, pathologic protein misfolding, and relative aggregation, novel therapies based on the supply of neuroprotective proteins have been proposed. Potential options for these protein-based treatments include proteases like Hsp70 and Hsp27 that can break down harmful protein aggregates, as well as prosurvival chaperone proteins like these [126]. Unfortunately, systemic injection drastically limits the amount of protein that can enter the brain due to the low permeability of the BBB. High doses and/or repeated treatments are required as a result, which raises side effects and decreases patient compliance [127]. The utilization of an inorganic clay nanocomposite system has been proposed as a means to enhance the inhibition of cholinesterase and improve the brain pharmacokinetics of donepezil [128].

## Metal based NPs

### Gold NPs (AuNPs)

Due to their usefulness, AuNPs have been the subject of extensive research. Because of its unique properties, gold can be easily manufactured in nanoscale sizes. Radiation therapy, thermal ablation, drug delivery, and medical diagnosis are only a few of the applications. Since it is challenging to transport therapeutic medications over the BBB in NDs, some studies evaluated the AuNPs in BBB models [129]. In an in vitro study, Ruff et al. analyzed the BBB-crossing potential of AuNPs paired with amyloid. The findings indicated that large or negatively charged NPs impede BBB crossing, but small AuNPs increase BBB integrity [130]. These results are consistent with those of another study that looked at smaller (20 nm) and larger (50 nm) insulin coated AuNPs in vivo. Maximum migration and accumulation of 20 nm AuNPs were seen in the brain, demonstrating the importance of nanoparticle size in the design of AuNPs for NDs. A number of studies have looked at AuNPs as potential drug delivery vehicles for brain-specific therapies [131]. Glutathione-encapsulated gold NPs have been developed to prevent the aggregation of Aβ in AD. These NPs penetrated the BBB and inhibited Aβ amyloid without causing any harm to the cells. This unique study shows how L and D chiral NPs have different effects, and it emphasizes how crucial stereochemistry is to the creation of nanoformulations [132]. Using rodents with AD induced by okadaic acid, additional research examined the antioxidant and anti-inflammatory properties of gold NPs. The reduction of oxidative stress and neuroinflammation caused by okadaic acid by AuNPs increases the likelihood that they could be effective as an AD treatment [133]. Additionally, AuNPs have been utilized to enhance the targeting of gene therapies for PD. Hu and colleagues produced AuNPs that can electrostatically bind to plasmid DNA. In both the in vivo PD model and in vitro transfection by endocytosis trials, effective transmission across the BBB was demonstrated. AuNPs can also be used to detect Aβ in AD. Based on the color shift of AuNPs, Zhou et al. developed a quantitative colorimetric sensor that can detect Aβ aggregation. The device is sensitive, inexpensive, and simple to operate [134].

### Silica nanoparticles (NPs)

According to studies, silica NPs are detrimental to neuronal cells, cause neurotoxicity, and result in neurodegeneration [135]. SiO2-NPs were administered intravenously to a mouse model, and cognitive impairment and an increase in anxiety were observed. In addition, exposure to NPs resulted in neurodegenerative symptoms, including neuroinflammation, increased phosphorylation of tau proteins, and deteriorated exocytosis. Mesoporous silica nanoparticles (MSNPs), a subclass of silica nanoparticles with advantageous properties such as a large surface area, better drug loading, and functionalization capabilities, have attracted interest. In an in vitro model, it was found that PEG-PEI functionalized MSNPs could cross the BBB without causing any negative side effects [136]. The synthesis and evaluation of silica NPs loaded with PEGylated quercetin were conducted in a model of copper-induced oxidative stress. The neural progenitors exhibited a heightened level of antioxidant activity when cultured in the brain. The neuroprotective properties of the substance suggest its potential utility in the therapeutic management of NDs [137]. To further improve BBB uptake, silica NPs were conjugated with a variety of ligands. Song et al. coupled the cationic iron-binding glycoprotein lactoferrin (Lf) with PEG silica NPs. This conjugation may be useful for nanocarriers for brain diseases, as it boosted the quantity of NPs transport over the BBB in the in vitro model. Similarly, Karimzadeh et al. created MSNPs that were functionalized with succinic anhydride and 3-aminopropyltriethoxysilane and supplied with rivastigmine hydrogen tartrate in a separate study. The formulation was effective in improving the drug’s stability and bioavailability; however, additional modification was suggested to reduce the viability loss caused by these NPs [138]. For the effective treatment of AD, brain-targeted lipid-coated MSNs containing berberine were synthesized in a separate study. The formulation exhibited a greater capacity to inhibit acetylcholine esterase (AChE). The research validated the inhibitory effects of lipid-coated MSNPs on amyloid fibrillation and malondialdehyde (MDA) levels, in addition to observing a substantial reduction in expression of beta-secretase 1 (BACE-1) levels [139].

### Cerium oxide NPs

Cerium oxide nanoparticles (CeONPs), commonly referred to as nanoceria, possess antioxidant and radical-scavenging properties that hold potential for therapeutic applications in the management of neurological disorders. Small CeONPs with a size of less than 5 nm can pass through the BBB. Brain cells can take in and store reactive nitrogen species to reduce them. In addition, they provide protection against mitochondrial fragmentation and neuronal cell death induced by AB [140]. Additionally, the neuroprotective properties of nanoceria were suggested as being advantageous for an AD model in humans. Cimini et al. conducted a study in which they observed the activation of the brain-derived neurotrophic factor (BDNF) signaling pathway upon the combination of polyethylene glycol (PEG)-coated CeONPs and an antibody targeting Aβ aggregates [141].

### Neural stem cell differentiation approach

Numerous findings have been published in recent years, encompassing the identification of adult neurons in the dentate gyrus of rats and the vocal control nucleus of birds. However, the understanding and interpretation of neurogenesis have undergone a recent shift. A major breakthrough was seen with the proliferation of progenitor cells and the subsequent creation of new neurons, which are dynamic processes. Age, alcohol use, and hormonal stress are just a few variables that may modify this process [142]. Researchers were also able to learn more about the cellular and biomolecular mechanisms included in adult neurogenesis by isolating, expanding, and differentiating neural precursor cells in an in vitro setting [143]. The discovery of neural stem cells (NSCs) and their function in the adult neurogenesis process prompted studies into NSCs’ regenerative abilities. A possible approach to treat various NDs is the development of therapeutics that can regulate the NSCs differentiation process and, consequently, the neurogenesis rate. Retrospective in vitro investigations served as the foundation for the recent concept of NSCs, which is associated with their unique biological traits. As per the provided definition, a NSCs refers to a type of stem cell that originates from various regions of the nervous system. These cells possess the ability to predominantly generate neurons, oligodendrocytes, and astrocytes when cultured in a controlled laboratory environment [144]. The tri-potent NSCs, predominantly observed in the subventricular zone and subgranular zone, are believed to be the source of adult neurogenesis [145]. After multiple investigations unequivocally showed that inhibiting neurogenesis reduced brain functioning while stimulating it led to a rebound in behavioral performance, such as learning and memory tasks, the therapeutic utility of NSCs was examined [146]. Therefore, the potential treatment strategies for NDs like AD and PD, which are typically characterized by declines in neurological function, may benefit from adult neurogenesis regulation. Despite the fact that the mechanism governing NSCs is controlled by a variety of physiological cues, NSCs is regarded as a key factor that affects neurogenesis. Adult neurogenesis was said to exist in both the brain’s subgranular zone (SGZ) and subventricular zone (SVZ). Some of the developmental stages involved are proliferation, differentiation, maturation, and integration. Each stage is distinguished by its own distinct cell phenotype. There is no standardized terminology to characterize the cells that support adult neurogenesis since SGZ and SVZ do not share identical progenitors. Numerous markers can be used to distinguish between the various cell phenotypes engaged in the process of neurogenesis [147, 148]. In the past 20 years, several techniques have been established to manufacture NSCs from pluripotent adult somatic cells or after they have been removed from niches and to differentiate them in vitro [149]. However, there is yet no defined standard strategy to stimulate NSCs differentiation *in* situ or in vivo. NSCs differentiation can be induced in a variety of ways, depending on whether the NSCs is endogenous or exogenous. Exogenous NSCs can be created by stimulating somatic cells or by being separated from neural niches (such as fibroblasts). Before being implanted into living beings, exogenous NSCs are cultured in vitro, and differentiation can be scheduled to take place either before or after cell culture. Endogenous NSCs can only differentiate in vivo and are confined to brain niches [150].

## Challenges

The inherent CNS intricacy still prevents the potential of various NSCs-based treatment approaches, despite significant and hopeful advances. Invasive methods are constrained by the CNS structural fragility, while the stage, location, and kind of pathology have a significant impact on the effectiveness of treatments. A notable constraint of NSCs research is the absence of a discernible correlation between the behavior observed in a controlled laboratory setting and that observed within a living organism [151]. The microenvironment affects NSCs capacity to differentiate into specialized lineages. The biggest obstacle to intensively controlling differentiation of NSCs in vivo is a lack of understanding of the chemical, physical, and cell-cell interactions. Controlling these cells biological activity and behavior after transplantation or stimulation is another difficulty [152]. In addition to this, limited information is available regarding the processes, the site, and the degree of the neurogenesis modulation in several therapeutic trials employing NSCs. The tight chemically defined conditions and potential for adaptive genetic modifications are additional issues with NSCs in vitro cultivation. Exogenous NSCs-based therapies are more challenging to develop since in vivo transplantation is typically accompanied by issues with cell source, cell survival, and graft rejection [153].

### Nanomedicine contribution to modulation of neural stem cell differentiation

**Fig. 4:**
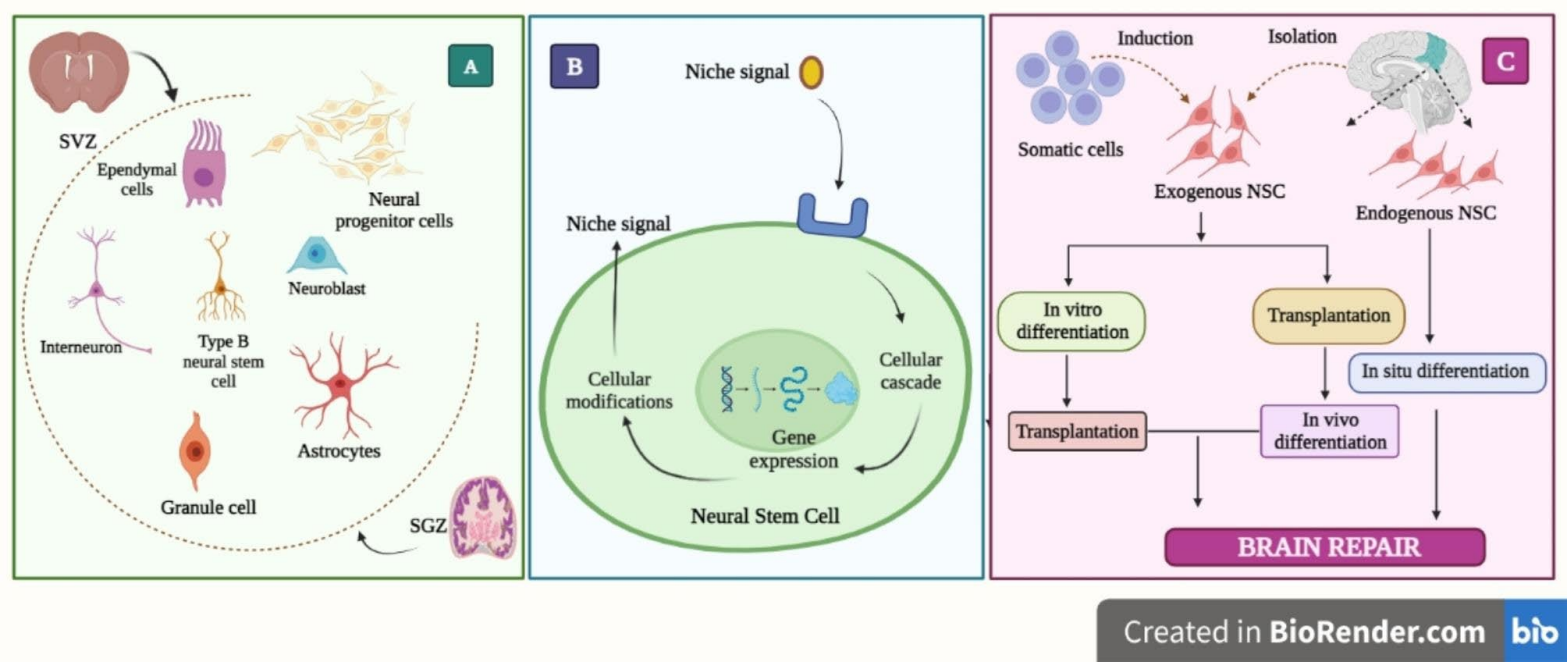
Localization and regulation of adult NSCs. (**A**) Depiction of various cells, including ependymal cells, NSCs, intermediate progenitor cells, neuroblasts, granule cells, and astrocytes, present in the subgranular and subventricular zones of the brain. (**B**) Extrinsic and intrinsic NSCs regulatory mechanisms are interconnected. Extrinsic signals can interact with NSCs plasma membrane receptors or enter the cell through certain channels, causing intracellular cascades that alter gene expression. By influencing intrinsic transcriptional programs, intrinsic regulatory pathways can also control the direction of gene expression in NSCs differentiation. **C**) Pathway involved in the differentiation of exogenous NSCs along with Pathway involved in the differentiation of endogenous NSCs.

The development of nanomedicines has addressed several limitations in the regulation of NSCs differentiation that are associated with conventional medicine. Several constraints include the lack of correlations between in vitro and in vivo NSCs behavior, unfavorable physicochemical profiles of drugs, and limited bioavailability [154]. One of the primary challenges associated with NSCs transplantation-based therapies pertains to the ability of the transplanted cells to effectively endure, undergo differentiation, and integrate into the pre-existing cellular network. Nanostructured scaffolds are attractive alternatives to simulate in vivo extracellular circumstances; the differentiation of NSCs after transplantation can be ensured by choosing the proper nanoscale material and architecture. For the in vivo transplantation of NSCs, nanostructured scaffolds have been produced. Landers et al. developed PLGA-based first carbon nanotube to trigger the differentiation mechanism of iPSC-derived NSCs and the formation of neural cells after the application of in vitro electric stimuli. The utilization of a nanostructured scaffold in patients with neurodegenerative disorders who are undergoing NSCs transplants holds promise as a potential strategy to improve cell viability and promote effective integration of these cells [155]. Illustration of the localization and regulation of adult NSCs is presented in (Fig. 4D).

**Table 1:** Nanomedicine-based systems for the regulation of NSCs differentiation

Nanomedicine delivery systems	Study model	Therapeutic outcomes	References
Porous silicon photonic crystals	In vitro	i-Differentiation of NSCs ii- improved neurogenesis and astrogenesis	[156]
Carbon nanotubes	In vitro	i-Activation of NSCs differentiation ii-enhancement of neuronal lineage expression iii-Controlled neuronal maturation	[155]
Neurogenin2-loaded nanoparticles	In vitro	i-Increased expression of neurofilament ii-maturation of NSCs	[157]
3D graphene oxide loaded gold nanoparticles	In vitro	i-Detection of NSCs differentiation ii-Understanding of survival of implanted NSCs	[158]
Polymeric nanovehicles loaded with retinoic acid	In vitro	i-Increased number of MAP-2 cells ii-Modulation of NSCs	[159]
miR-124-nanoparticles	In vivo (intracerebral)	i-Modulation of neurogenic niche ii- Recovery of PD deficit	[160]
Poly-L-lysine-Fe2O3 decorated nanoparticles	In vivo	i-Magnetic resonance imaging of iPSC-derived NSCs ii-Monitoring of proliferation and differentiation of NSCs	[161]
Salmon fibrin nanofibers	In vivo	i-NSCs proliferation and differentiation ii-Enhanced vascularization	[162]
Self-assembled peptide PCL-PLGA nanofibers	In vivo	i-Differentiation and proliferation of NSCs in oligodendrocytes and neurons ii-Improved regeneration of nervous system	[163]

Nano biomaterials-based systems for the regulation of NSCs differentiation are tabulated in Table 1. Nanomedicine offers non-viral vectors for cell reprogramming, such as nanoparticle-based methods. Li et al. used a biodegradable nanoparticle-mediated transfection technique to stimulate mature neuron development. They implanted human fetal tissue-derived NSCs at the target site in the brain of a rat and administered neurogenin, which led to a much higher production of neurofilament [157]. According to research, in a rat model of AD, functional deficits are recovered after the administration of curcumin-loaded NPs, which regulate and control NSCs differentiation. Learning and memory deficits were improved after these NPs were injected intraperitoneally and boosted the expression of genes related to brain development [103]. One of the most effective strategies for treating neurodegenerative illnesses is in situ NSCs differentiation. However, no research employing this paradigm has yet progressed to the clinical stage. Non-selective systems are primarily encouraged by the absence of NSCs-targeting drugs. Only a small number of nanoparticulate delivery technologies have been developed that deliver active compounds to the neurogenic region and specifically target endogenous NSCs. More recently, NFL-decorated lipid based NPs that target specifically SVZ-NSCs in vitro as well as in vivo following intracranial injection were developed by Carradori et al. [164]. A viable approach to addressing issues with transplantation is the development of technology that can target endogenous NSCs. The possibility of the transplanted NSCs dying or being rejected would be completely eliminated. Endogenous NSCs targeting may also increase the therapy effectiveness by increasing drug absorption and thereby reducing side effects.

### Gene therapy

**Fig. 5:**
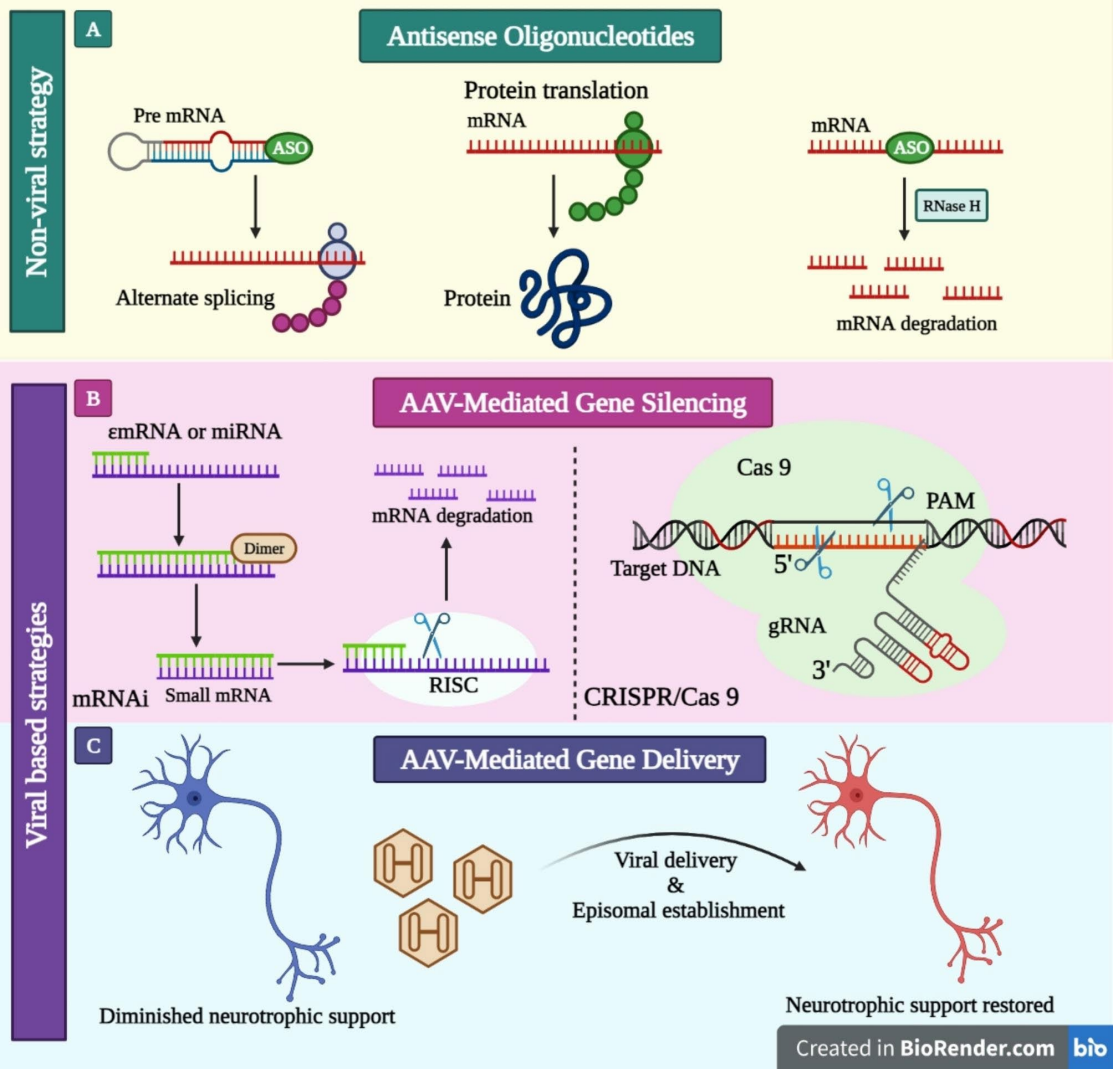
Currently available gene therapy vectors; (**A**) ASOs are used in non-viral approaches to trigger RNase H-mediated degradation or alternative splicing. (**B**) RNA interference and CRISPR-Cas9-mediated AAV-mediated gene silencing are examples of viral techniques for gene manipulation. (**C**) Transduction of neurotrophic factors into genes via AAV-mediated gene delivery

Gene therapy has the potential to provide therapeutic advantages to a significant number of individuals suffering from neurodegenerative conditions. This can be achieved through various means, such as directly addressing the underlying pathological mechanisms, offering neuroprotection, facilitating neurorestoration, and effectively managing symptoms [165]. Gene therapy is emerging as a promising treatment option for various NDs, including AD, PD, and HD [166]. The transfer of a transgene that either corrects or substitutes a faulty gene or generally maintains cells in the illness environment is used to treat disease. In actuality, it is far more complicated, and several variables need to be optimized. The transgene must be chosen, the proper vector must be selected, and the delivery method must be optimised. The host immune system’s interaction with the vector or transgene may make treatment even more challenging. The characteristics of the target tissue provide an additional level of difficulty for NDs [167]. The primary advantage of this technology lies in its ability to offer a potentially enduring therapeutic impact without necessitating repeated administration. There are currently available gene therapy vectors that can be categorized into two main types: viral vectors and non-viral vectors ((Fig. 5E)). Viral vectors such as vaccinia, measles, vesicular stomatitis virus (VSV), polio, reovirus, adenovirus, lentivirus, retrovirus, herpes simplex virus (HSV), and adeno-associated virus (AAV) have been among the viral vector types that have been utilized in clinical trials [168].

### Vectors used in gene therapy

**Retroviral vectors**: These are the encapsulated RNA viruses that integrate into the genome of the host cell through reverse transcription in DNA within the host cell. In a process known as pseudotyping, RV can adopt a surface protein from any other virus to acquire specificity. Pseudotyping facilitates the transduction of several CNS area cells by vectors. **Lentiviral vector (LV)**: The Retroviridae family encompasses LV vectors, which are derived from the human immunodeficiency virus (HIV-1). These vectors play a crucial role in maintaining gene expression in the brain over an extended period of time, as they have the ability to transduce both quiescent and proliferative cells, including neurons. **Adenoviral vector (AV)**: Both quiescent and proliferating cells respond favorably to AV43. The transgene may be expressed at a high level in AV, which also has a larger spectrum of tropism and strong transduction potential. Despite its many advantages, AV has certain drawbacks, such as its high immunogenicity, potential for cellular reactions, and transient gene expression [169].

### Features and delivery of viral vectors

The process of introducing a transgene into a vector is a multifaceted procedure, necessitating the presence of certain notable characteristics in the vectors. Specifically, the vector must facilitate straightforward modification of recombinant technology and allow for replication in suitable host organisms. The vector should have a high capacity for cloning and little invasiveness. The chosen transgene must only express itself in the target cells. It should not produce any immune responses. The vector must enable stable, extended production of a functioning gene without affecting cell offspring. Viral vectors may be directly and effectively distributed throughout the whole brain via convection-enhanced delivery (CED). In order to transport huge volumes of highly concentrated macromolecules, CED relies on fluid convection rather than passive diffusion. The process utilizes a pressure gradient to induce turbid flow within the interstitial fluid compartment [170].

## Strategies of gene therapy

### Antisense strategy

The application of the antisense technique in gene therapy involves the utilization of substances that regulate cellular mechanisms responsible for genetic information processing, specifically in instances characterized by genetic abnormalities. By interfering with transcription or translation, the antisense method aims to prevent the production of the target protein in the cell. The need for methods to specifically stop the manufacture of toxic proteins in the brain is highlighted by our growing understanding of the role that unfavorable gene expression patterns play in the development of neurodegenerative disease. Antisense technologies are perfectly suited for this use case [171]. Modified nucleic acids, known as antisense oligonucleotides (ASOs), exhibit the ability to bind to RNA molecules through the process of Watson-Crick base pairing, thereby exerting regulatory control over their functional activities. The binding of ASO molecules can lead to either gene silencing or modification of RNA metabolism, depending on the specific chemistry and sequence involved. ASO-mediated gene silencing employs two distinct mechanisms: degradative and non-degradative. Gene silencing can be achieved through two main mechanisms: degradative processes and non-degradative mechanisms. In degradative processes, the target RNA is cleaved by naturally occurring nucleases. On the other hand, non-degradative mechanisms involve the binding of ASOs to hinder translation, 5′ capping, or splicing, thereby preventing gene expression [172]. The prevailing approach in the development of ASO medications involves the utilization of a degradative mechanism, whereby RNase H is attracted to the oligonucleotide-RNA heteroduplex. The catalytic activity of a single ASO can initiate the degradation of multiple RNA molecules. This occurs when the enzyme RNase H recognizes and cleaves RNA molecules that are hybridized to DNA in a heteroduplex structure [173].

### RNA trans-splicing

RNA trans-splicing is a molecular process that facilitates the substitution of a mutated segment of pre-mRNA with a healthy sequence capable of encoding functional proteins. Additionally, it enables the fusion of distinct pre-mRNA molecules, resulting in the formation of composite mRNA. Further investigation is required to ascertain the therapeutic viability of RNA trans-splicing in the context of neurodegenerative disorders. The utilization of spliceosome-mediated RNA transsplicing, commonly referred to as SMaRT, exhibits considerable potential in the development of innovative gene therapy interventions targeting hereditary diseases that currently lack effective treatment options. The SMaRT technique operates by altering the mRNA sequence, thereby leveraging the repair mechanisms to correct mutations occurring at the post-transcriptional stage. In order to induce a splice event in Trans between the target endogenous pre-mRNA and exogenous RNA, a common approach involves exposing the target cell to exogenous RNA through gene transfer. As a consequence, chimeric mRNA is generated, comprising exonic regions derived from both the latter and former sequences and encoding a sequence devoid of mutations [174]. The two most common forms of spliceosomal trans-splicing are genic trans-splicing and spliced leader (SL) trans-splicing. Genetic trans-splicing is the process of joining together pieces of two different RNA transcripts via splice sites. These exons can originate from different mRNA precursors within the same gene, different gene transcripts, or even intergenic regions transcribed from other chromosomes. Generically, various trans-splicing choices considerably expand the repertoire of RNA transcripts, potentially increasing proteome complexity. During SL trans-splicing, a tiny exon is moved from the 5′ end of a larger, more specialized RNA molecule to the 5′ end of a messenger RNA molecule, where it serves as the first exon of the message. Multiple distinct transcripts can be joined together using SL exons to produce mature mRNAs that share a 5’-terminal sequence [175].

### RNA interference (RNAi)

RNA interference is an approach to regulating viral replication and gene expression. Both methods of silencing genes, RNA interference and gene silencing, take advantage of double-stranded RNA. According to the sequence, double-stranded RNAs generate small interfering RNAs inside the cell that can identify and destroy complementary RNAs. When a sequence homology is established between a small interfering RNA (siRNA) and a specific mRNA molecule, the siRNA activates cellular nucleases, resulting in a decrease in the mRNA level. Therefore, siRNA can be used to mute specific genes involved in the pathophysiology of a number of diseases that have been shown to have a genetic basis. In the absence of a definitive treatment for neurodegenerative diseases, siRNA offers hope for the development of novel therapeutic strategies [176]. The machinery required for RNA interference is present in all eukaryotic cells. First, double-stranded RNA precursors are “sliced” by the RNAse III-like enzyme Dicer into siRNAs with a length of 21–23 nucleotides. When these siRNAs are mixed with a protein complex, a silencing complex known as the RNA-induced silencing complex (RISC) is formed [177]. RISC can only identify the matching mRNA if one of the strands remains linked to the complex. Using this strategy, RNAi achieves its high degree of sequence specificity. One of the key proteins in RISC, Argonaute-2, then cleaves the homologous mRNA. RNAi is highly efficient because a single RISC complex can destroy several messenger RNAs [178].

### Phytochemicals and their use in degenerative disorders

#### Phytochemicals

The incidences of neuro-degenerative disorders are increasing at an alarming rate, with no effective treatment, as the world progresses, as a result of recent lifestyle choices and a large number of elderly people. Recent research suggests that phytochemicals and polynutrients will be used to treat neurological disorders [179]. Phytochemicals are typically secondary metabolites that play no significant role in plant growth or development. According to studies, these secondary metabolites are quite advantageous for human health and disease prevention. Phytochemicals’ diverse chemical structures including alkaloids, saponins, indoles, phytosterols, and isothiocyanates all contribute to their ability to benefit human health. These positive outcomes arise from a number of processes, including free radical scavenging, blocking the construction of microfilaments and microtubules, and inhibiting proteases [180].

#### Phytochemicals and BBB

The therapeutic effects of phytochemicals are attributed to their capacity to traverse the BBB and their dosage. The BBB restricts the passage of various substances, including phytochemicals, into the brain. However, there exists a finite subset of phytochemicals that possess the ability to traverse the BBB, regardless of whether their dosage exceeds or falls below 10 mg/kg/day. Several candidates have been identified to exhibit a neuroprotective effect, one of which is icariin. Icariin, derived from Herba Epimedii, has been found to demonstrate therapeutic efficacy in a mouse model of PD. Dopaminergic neurons were protected by the neuroprotective properties of Icariin. When 2–6 mg/kg/day of icariin was administered to Sprague-Dawley rats over a four-month period, autophagy-related proteins were upregulated in the cortex and hippocampus of geriatric rats [181]. One study showed that E. alsinoides and C. asiatica ethanolic extracts reduced scopolamine-induced cognitive deficits in rats. This impact is partly due to the restoration of brain antioxidant enzyme activity, suppression of inflammatory indicators, and inhibition of AChE, which scopolamine negatively affects [182]. Numerous phytochemicals, such as resveratrol, quercetin, and catechins, are associated with neuroprotective effects. However, the complex chemical structures of phytochemicals make them relatively impermeable to the BBB, thereby limiting their ability to treat brain disorders [183]. The treatment of NDs using phytochemicals faces various challenges beyond the BBB. These obstacles encompass the blood vessels, gastrointestinal tract (GIT), and mucosa, as well as the potential loss of phytochemicals during the initial passage through metabolism, primarily caused by enzymatic degradation [184]. To address this obstacle, researchers have extensively used nanocarriers based drug delivery strategies. Phytochemicals, which encompass hydrophilic and lipophilic molecules, can be effectively delivered by encapsulating and conjugating them with nanocarriers [185]. The choice of nanocarrier material is crucial as it determines the bio-efficacy of the delivery system [186]. Moreover, these nanocarriers exhibit a higher degree of target specificity, enhancing their therapeutic potential.(Fig. 5F) exhibit schematic illustration of the mechanisms by which phytoconstituents reverse the symptoms of NDs. The field of neurological science has experienced significant impact as a result of the advancements in nanotechnology [187]. Recent advancements in the field of nanotechnology have opened new possibilities for the development of innovative therapeutic drugs targeting brain disorders. In contemporary times, there has been significant progress in the development of nanoparticles containing phytochemicals that fall within the size range of proteins. These nanoparticles have the ability to traverse the BBB and exhibit their respective functions within the brain [188].

**Fig. 6:**
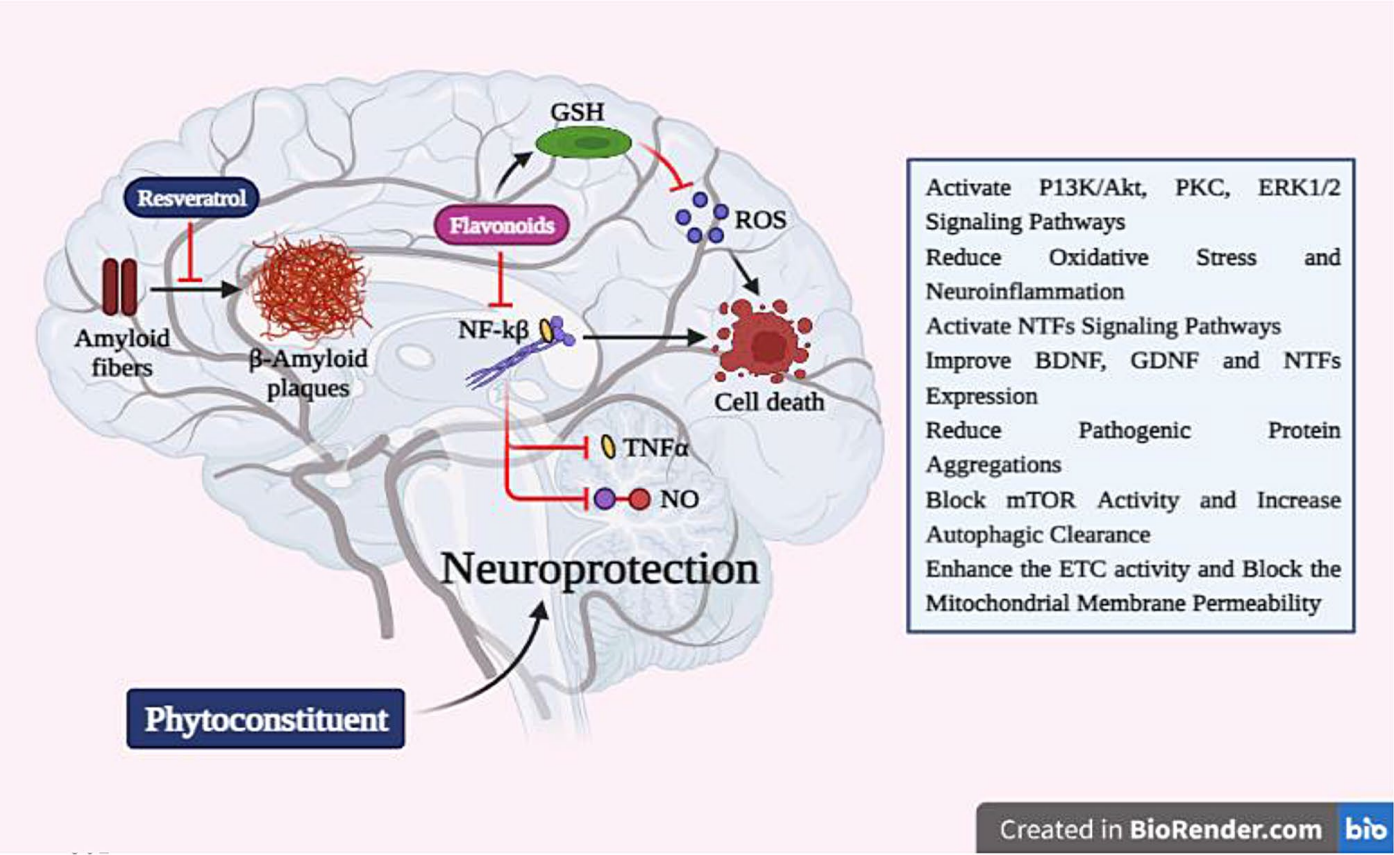
A schematic illustration of the mechanisms by which phytoconstituents reverse the symptoms of NDs. Phytochemicals exert their effects on various targets, such as the inducible nitric oxide synthase (iNOS) and the ratio of BCL-2/BAX, which facilitates the restoration of mitochondrial function; stimulation of the P13K/Akt, PKC, signaling pathway; enhancement of the expression of BNDF, GNDF, and NTFs; augmentation of the activity of SOD, Catalase, and GSH to safeguard neurons against oxidative stress-induced damage; inhibition of Lewy body formation; and protection of neurons by directing the anti-inflammatory pathway; and inhibiting the transcription factor NFκB. The protective effects of phytochemicals on neurons are pervasive

#### Recent uses of phyto-nanomedicine for NDs

NDs exhibit a strong correlation with advanced age and have demonstrated a persistent prevalence due to the escalating proportion of elderly individuals within the population in recent times. However, the primary limitation lies not in the mere occurrence of the disease, but rather in the lack of efficacy exhibited by the treatment methods employed. Synthetic drugs are deemed unsuitable for routine therapeutic use due to the adverse effects they elicit. In order to circumvent the utilization of synthetic drugs, researchers have directed their attention towards phytochemicals possessing specific attributes such as antioxidant, anti-inflammatory, anticholinesterase, and anti-amyloid properties in their natural form [189]. These phytochemicals exhibit considerable potential as therapeutically active agents for the treatment of neurodegenerative disorders [190]. Nanotechnology has emerged as a promising avenue for drug development, offering new possibilities in the field. However, it is significant to observe that the application of nanotechnology in theranostic approaches for diseases such as AD and PD remains limited at present. NPs present certain limitations, such as their potential for toxicity and their relatively high cost [191]. Research findings have indicated that the chemicals used in the production of NPs have a propensity to adhere to the surface of these particles, rendering them unsuitable for medical applications. Recent research studies have demonstrated that nanoparticles incorporating phytochemicals exhibit noteworthy outcomes. The neuroprotective effects of naturally occurring polyphenol EGCG-coated selenium nanoparticles have been observed in various studies. Epigallocatechin gallate (EGCG) has been found to exhibit inhibitory effects against amyloid beta, transthyretin, α-synuclein, and huntingtin. These proteins are widely recognized as the primary contributors to the progression of AD [192]. Ajugarin-I, a phytoconstituent, has been found to possess neuroprotective properties against vincristine-induced neuropathic pain. The potential mechanism underlying the anti-neuropathic effect of Aju-I may involve the activation of the nuclear factor erythroid 2-related factor 2/heme oxygenase 1 (Nrf2-HO-1 axis), the inhibition of Nuclear factor-kappaB (NF-kappaB)/cyclooxygenase-2 (NF-κB/COX-2), and apoptotic signalling in nociceptors located in the spinal cord [193]. This section presents a comprehensive analysis of nanocarrier systems that incorporate phytoconstituents or phytochemicals for the purpose of neuroprotection against NDs. The subsequent table showcases specific examples of these nanocarriers systems (Table 2).

**Table 2:** Phytochemical-loaded nanocarriers for the treatment of NDs

Phytochemicals	Nanocarriers	Therapeutic activity	Study outcomes	References
Curcumin	Polymeric NPs	Anti-amyloid activity, antioxidant activity	Reduced aggregation of amyloid plaques. Reduced oxidative stress.	[102]
Resveratrol	Solid Lipid NPs	Anti-amyloid activity, reduce oxidative stress	Inhibits Aβ formation in the brain. Increase BBB permeability.	[194]
Epigallocatechin-3-gallate	Polymeric NPs	TAU-aggregation inhibitors, anti- amyloid activity	Increase drug permanence in the brain. Reduced neuroinflammation.	[195]
Catechin	Liposomes	Antioxidant, anticholinesterase, anti-amyloid aggregation, anti-inflammatory activity	Reduced oxidative stress, reduced neuroinflammation.	[196]
Quercetin	Liposomes	Anti-amyloid, anti-cholinesterase activity. Improves cognitive and memory impairment	Effective permeation of quercetin across BBB. Improved oral bioavailability.	[197]
Thymoquinone	Solid lipid NPs	Anti-amyloid activity	Efficiently targets the brain and delivers the drug in a controlled manner. Decreased neuroinflammation.	[198]
Sesamol	Solid lipid NPs	Neuroprotective, repairs learning and memory impairment	Improved cognitive functioning.	[199]
Naringenin	Nanoemulsions	Anti-amyloid activity	Reduced Aβ toxicity in brain.	[199]
Tannin	Microemulsions	Anticholinesterase, antioxidant activity	Enhance Ach levels, decrease cytokines level for oxidative stress.	[200]
Ferulic acid	Chitosan coated solid-liquid NPs	Reduce ROS production, anti-inflammatory activity	Improved permeability across BBB.	[201]
Camptothecin	Solid lipid NPs	Anti-amyloid activity	Enhanced Penetration through BBB.	[202]
Ginkgolic acid	Polymeric NPs	Neuroprotective, reduced ROS production	Improved oral bioavailability.	[203]
Ethanoic bark extract	Gold NPs	Neuroprotective, antioxidant and anticholinesterase activity	Reduced oxidative stress and inflammation.	[204]
Hesperetin	Nanocrystal	Antioxidant activity.	Improved cognitive functioning.	[205]
Morin hydrate	Micellar nanocarriers	Restore cognitive functioning	Improvement in memory and cognitive functioning.	[206]
Leaf extract of Mentha piperita	Silver NPs	Anticholinesterase activity	Improve permeation through BBB and reduced Aβ toxicity.	[207]

#### Nanomedicines in research for the treatment of NDs

The utilization of nanomedicines in the realm of scientific research has garnered significant attention as a potential avenue for the treatment of NDs. Table 3 describe the recently developed nano formulations for the treatment of NDs. These nanomaterials based formulation have showed remarkable therapeutic outcomes including better transport to the brain with targeting effect. Moreover, inhibition of Aβ aggregation, abridged brain damage, enhanced neuroprotection and behavioral improvement in the NDs was observed in preclinical studies.

**Table 3:** Selected pertinent preclinical investigations grounded on recent nanoformulations for the treatment of NDs

Encapsulated molecules	Type of Nanocarriers	Therapeutic outcomes	References
Tacrolimus and rapamycin	Liposomes	Improved graft survival and fiber expansion in dopaminergic neurons for better control of NDs.	[208]
Clioquinol	Metal nanoparticles (Mesoporous silica nanoparticle with a gold cap)	Release of CQ and AuNPs upon exposure to high H2O2 levels resulting in inhibition of Cu2+-induced Aβ aggregation. Reduced rupture of the cell membrane, microtubular abnormalities, and ROS-mediated apoptosis caused by Aβ 40-Cu2 + complexes.	[209]
Curcumin	Lipid-based nanoparticles	Reduced brain damage and slowed neuronal loss via multiple mechanisms in ALS, HD, PD, and AD.	[210]
Activin B	Hydrogel	Sustained release of activin for enhanced neuroprotection, considerable cellular protection, and behavioral improvement in PD.	[211]
Curcumin	Polymeric nanoparticles	Increased BBB permeability significantly reduced Aβ aggregates. Improved drug delivery, minimized oxidative stress and inflammation.	[212]
Cholesterol-bearing pullulan, CHP	Nanogel	Minimized Aβ aggregation. Inhibition of formation of Aβ fibrils.	[213]
Ginkgolide B	Polymeric nanoparticles	Sustained release of GB, Enhanced accumulation in the brain for effective PD treatment.	[214]
Phytol	Polymeric nanoparticles	Extended lifespan, enhanced chemotaxis behavior, reduced Aβ deposition. Decreased production of ROS with improved neuroprotective efficacy in in vivo model of AD.	[215]
Berberine and Curcumin	Transferosomes	Sustained release of both drugs, Intranasal administration improved spatial memory in AD. Antioxidant activity enhanced inhibition of Aβ fibrils formation and reduced the regulation of BACE-1 expression.	[216, 217]
Donepezil	Dendrimer	Enhanced half-life of donepezil resulting in higher brain absorption. Improved inhibition of AChE in vitro, enhanced transport to the brain in vivo.	[218]

#### Clinical trials —update

The website clinicaltrials.gov is a comprehensive platform that serves as a valuable resource for accessing information related to clinical trials. It presents a considerable volume of information that has the potential to yield valuable insights into the present status of clinical research concerning diverse diseases. In this study, a meticulous and methodical approach was employed to organize and analyze data pertaining to clinical trials conducted for NDs (Table 4). The objective was to generate succinct and informative tables that effectively communicate key details about these trials, including their respective clinical phases and types of approaches employed. The application of this methodology has enabled the identification of noteworthy trends in clinical trials over time regarding the intervention mechanism in therapy, investigation of different “Biologic” medications, and the development of drug delivery systems. In recent years, the scientific community has made notable progress in the field of managing symptoms related to NDs. However, there is an increasing emphasis on therapeutic innovation with the objective of targeting the fundamental mechanisms responsible for the progression of these diseases.

**Table 4:** Detailed description of the ongoing clinical trials and the approved/marketed products for the treatment of NDs

Products	Sponsor/ Organization	Target disease	Approach	Clinical trial.gov ID	Status
Donepezil	Eisai, Inc.	AD	cholinesterase inhibitors	----------	Approved
Memantine	Eli Lilly/ Reddy’s Laboratories	AD	Cholinesterase inhibitors and regulators of glutamate receptors	----------	Approved
Donanemab	Eli Lilly & Co	AD	Inhibit Aβ aggregate formation	----------	Request for complete approval
Leqembi	BioArctic AB, Biogen, Eisai	AD	Inhibit Aβ aggregate formation	----------	Complete approval
Aduhelm	Biogen, Neurimmune	AD	Inhibit Aβ aggregate formation	----------	Expedited approval
Donanemab|/Aducanumab (TRAILBLAZER-ALZ 4)	Eli Lilly & Co	AD	Passive immunotherapy	NCT05108922	Active, not recruiting (Phase III)
MSC-Exos	Cell Biomedicine Group	AD	Cell therapy	NCT04388982	Phase II
Lecanemab and E2814 DIAN-TU-001 (E2814)	Eisai	AD	Passive immunotherapy	NCT05269394	Recruiting (Phase III)
NE3107	BioVie	AD	Anti inflammatory	NCT04669028	Phase III
Remternetug (TRAILRUNNER-ALZ1)	Eli Lilly & Co	AD	Passive immunotherapy	NCT05463731	Recruiting (Phase III)
ALZ-801 (APOLLOE4)	Alzheon	AD	Inhibit Aβ aggregate formation	NCT04770220	Active, not recruiting (Phase III)
Simufilam (RETHINK-ALZ)	Cassava Sciences	AD	Disrupting the linkage of FLNA with α7nAChR to prevent toxic Aβ fibrils formation	NCT04994483	Recruiting (Phase III)
CT1812 (SHINE (COG0201)	Cognition Therapeutics	AD	displacement of Aβ oligomers	NCT03507790	Recruiting (Phase II)
ACI-35	AC Immune SA	AD	Passive immunotherapy	NCT04445831	Active, not recruiting (Phase II)
LX1001	Lexeo Therapeutics	AD	Gene therapy	NCT03634007	Phase I
Carbidopa-levodopa	Merck	PD	DOPA decarboxylase inhibitor/DA precursor	----------	Approved
Ropinirole	GlaxoSmithKline Pharmaceuticals.	PD	DA agonist	----------	Approved
UB-312	Vaxxinity	PD	Peptide based therapy Targets alpha-synuclein	NCT04075318	Phase I
Prasinezumab	Hoffmann-La Roche	PD	Humanized IgG1 based therapy Targets aggregated alpha-synuclein	NCT03100149	Phase II
IRLAB’s mesdopetam	Therapeutics and Celon Pharma	PD	Targets D3 receptors	NCT04435431	placebo-controlled Phase II trial
AAV2-GDNF	Brain Neurotherapy Bio, Inc.	PD	Adeno-associated virus delivery of neurotrophic factor	NCT04167540	Phase I
Aptinyx’s NYX-458	Aptinyx	PD	Targets NMDA receptors	NCT04148391	placebo-controlled Phase II trial
Celon’s	Therapeutics and Celon Pharma	PD	Targets phosphodiesterase 10 A (PDE10A)	NCT05297201	placebo-controlled Phase II trial
IPX-203	Amneal Pharmaceuticals	PD	Extended-release version of CD/LD to reduce systemic fluctuation	NCT03877510	Phase III trial
Riluzole	Covis Pharma/ Sanofi	ALS	Excitotoxicity	----------	Approved
Edaravone	Mitsubishi Tanabe Pharma	ALS	Oxidative stress	----------	Approved
Memantine	MND-SMART	ALS	Excitotoxicity	NCT04302870	Phase II/III (removed from trial in April 2023)
Trazodone	MND-SMART	ALS	Oxidative stress	NCT04302870	Phase III (removed from trial in April 2023)
Ravulizumab	Alexion Pharmaceuticals, Inc.	ALS	Neuroinflammation	NCT04248465	Stopped in January 2023
Lenzumestrocel	Corestemchemon, Inc.	ALS	Stem cell therapy	NCT04745299	Phase III
Jacifusen/ION363	Ionis Pharmaceuticals, Inc.	ALS	Gene specific–ASO, FUS-mutations	NCT04768972	Phase III
Tominersen	Hoffmann-La Roche	ALS	Antisense therapy	NCT02519036	Phase III
Risperidone	Janssen Pharmaceuticals, Inc.	HD	Inhibit 5-HT and D2 receptors	----------	Approved
Cellavita HD	Azidus Brasil	HD	Stem cell therapy	NCT02728115	Phase I
AMT-130	UniQure Biopharma B.V.	HD	Gene therapy	NCT04120493	Phase I/II (1b/2a clinical trials)
Cannabidol	Fundacion para la Investigacion Biomedica del Hospital Universitario Ramon y Cajal	HD	Anti inflammatory	NCT01502046	Controlled Phase II Clinical Trial
Fenofibrate	University of California, Irvine	HD	Target proliferator-activated receptor (PPAR)	NCT03515213	Phase II a

### Nanomedicines under clinical trials

A limited number of studies investigating the application of nanoparticles in the treatment of neurological disorders were identified. According to a recent search of ongoing clinical studies, Table 5 presents a compilation of nanoparticle-based formulations that are currently being investigated in clinical trials for the treatment of neurodegenerative disorders (NDs), with the number of formulations listed being less than ten. The utilization of a lipid nanoparticle-based formulation in the treatment of transthyretin-mediated amyloidosis has been investigated in a solitary clinical trial, which yielded positive outcomes and subsequently received regulatory permission for commercial distribution. The ongoing clinical trials involve a study utilizing CRISPR/Cas9 gene technology, which is now in its Phase I. In this investigation, lipid nanoparticles are being employed as a means of delivering the therapeutic medication.

One study titled “31-MRS Imaging to Evaluate the Impact of CNM-Au8 on the Altered Neuronal Redox State in Parkinson’s disease” investigates the influence of gold nanocrystals on individuals with PD by analysing their pharmacokinetic and pharmacodynamic properties, as well as assessing their safety [219]. Second research is being conducted with the working title “Study of APH-1105 in Patients with Mild to Moderate Alzheimer’s Disease” to evaluate the safety and efficacy of APH-1105 when administered via the nasal route for the potential treatment of AD.

**Table 5:** List of the nano biomaterials based products under clinical trials for the treatment of NDs

Product	Nanocarrier	Sponsor/ Organization	Target disease	Clinical trial.gov ID	Status
APH-1105	Nanoparticle	Clene Nanomedicine	AD	NCT03806478	Phase II
CNM-Au8 (Nanocrystalline gold)	Gold nanocrystals	Clene Nanomedicine	ALS	NCT04081714	Phase I
CNM-Au8 (Nanocrystalline gold)	Gold nanocrystals	Clene Nanomedicine	ALS	NCT04098406	Phase II
ACI-35.030 & JACI-35.054	Nanoparticle (Liposomes)	AC Immune SA	AD	NCT04445831	Phase II
CNM-Au8 (Nanocrystalline gold)	Gold nanocrystals	Clene Nanomedicine	PD	NCT03815916	Phase II

Although nanotechnology has been developed for many years, the first wave of applications has only recently begun. Current publications have depicted great interest in the ability of packaging multiple medications or biomolecules in nanostructures to alter neuronal regeneration, prevent microbial infections, or anti-inflammatory activities within the CNS. All together, these results provide a crucial framework for future research into pharmacokinetics improvement, system medication toxicity reduction, and early illness detection in humans. These developments cannot come fast enough, given the danger of a considerable rise in the incidence of neurological disorders [220].

### Future perspectives

Current therapeutic methods fail to effectively halt the advancement of NDs because of the physiological aspects and intricate pathways involved. Despite numerous research efforts, therapy outcomes continue to be inadequate, revealing a lack of congruence between research outputs and their testing in clinical trials. Huge amounts of scientific research in the past few decades have found a plethora of novel ways to administer CNS drugs. Many of the systems have extremely interesting medicinal applications. Despite the availability of medications that claim to alleviate or prevent NDs symptoms, the disease continues to advance. However, a variety of approaches emphasize the inherent difficulties in therapeutic and imaging agent transport across the BBB. Various medications, biomolecules (proteins, peptides, mAbs) and phytoconstituents have been utilized as therapeutic agents for the administration of NDs in an effort to discover more effective treatment methods. Another strategy to treating NDs involves the utilization of gene therapy, which entails comprehending the specific form of NDs and the genes implicated in its progression. One prospective approach for tackling numerous neurological illnesses entails the utilization of NSCs and the development of therapeutic interventions capable of modulating the differentiation process of NSCs, hence exerting an influence on the pace of neurogenesis. Although a treatment plan may not be created immediately, such study lays the groundwork for a strategy that would help millions of people worldwide live healthy lives and eliminate brain damage-related diseases. Nanomedicine and neurobiology may create new treatments for several NDs. Nanomedicine faces toxicological problems, yet biocompatible nanocarriers can overcome them. Nanomedicine’s persistent toxicity must be studied for clinical use. Nanotechnology as a typical NDs treatment is also expensive and thus new ways must be found to prepare cost effective treatment modalities with improved safety and clinical efficacy.

## Conclusions

Despite the availability of numerous active pharmaceutical agents to treat NDs, many of them are unable to cross the BBB to produce remarkable results in clinical trials. Nanomedicine is an emerging technique with the potential to eliminate the obstacles associated with the traditional medicine. Nano biomaterials based brain targeting strategies appears to be the alternatives being considered for addressing these issues, as it enables the modification of specific neural pathways in explicit brain regions and produce desired therapeutic effects by targeting the particular brain regions. Similarly, the integration of gene therapy and nanotechnology has the potential to enhance the effectiveness of gene therapy in the treatment of NDs. In addition, nano biomaterials oriented methodologies have lately enhanced the efficacy of the current neural stem cells (NSCs) differentiation-based therapies. Moreover, it helped in understanding the molecular foundations of the process involved. The development of NSCs-targeting systems may be one of the safest and most efficient approaches in NDs. It can be concluded that the utilization of nanocarriers, phytoconstituents, gene therapy, and NSCs approaches are the diverse therapeutic strategies aimed at treating NDs.

## Data Availability

All the data reported in this manuscript is available within the text.

## Abbreviations

NDs: Neurodegenerative diseases
CNS: Central nervous system
BBB: Blood-brain barrier
CSF: Cerebrospinal fluid
BCSF: The Blood cerebrospinal fluid barrier
AD: Alzheimer’s disease
PD: Parkinson’s disease
HD: Huntington’s disease
ALS: Amyotrophic lateral sclerosis
COMT: Catechol-o-methyltransferase inhibitors
MAO: Monoamine oxidase inhibitors
BMVECs: Brain microvessel endothelial cells
NPs: Nanoparticles
TNF-α: Tumor necrosis factor alpha
NGF: Nerve growth factor
RNAi: RNA interference
SOD1: Superoxide dismutase 1
TARDBP: TAR DNA-binding protein 43
FUS: Fused in sarcoma
TBK1: TANK-binding kinase 1
fALS: Familial amyotrophic lateral sclerosis
C9orf72: Chromosome 9 open reading frame 72
sALS: Sporadic ALS
P-gp: P-glycoprotein
RME: Receptor-mediated endocytosis
TMT: Transporter-mediated transcytosis
AMT: Absorptive-mediated transcytosis
NMs: Nanomedicines
mAbs: Monoclonal antibodies
Tf: Transferrin
TfR: Transferrin receptor
LDL: Low-density lipoprotein
apoB-100: Apoprotein B100
apoE: Apolipoprotein E
InsR: Insulin receptor
LDLR: Low-density lipoprotein receptor
APP: Amyloid precursor protein
GLUT: Glucose transporters
LNPs: Lipid-based nanoparticles
SLNs: Solid lipid nanoparticles
NLCs: Nanostructured lipid carriers
EPO: Erythropoietin
AB: Amyloid beta
hCMEC/D3: Human cerebral microvascular endothelial cell line
EGCG: Epigallocatechin-3-gallate
PECA: Poly ethyl cyanoacrylate
PEG-PEI: Polyethylene glycol with polyethyleneimine
HA: Hylauronic acid
A 1–28: 28-amino-acid AB peptide
dPG: Dendritic polyglycerol derivatives
HSA: Human serum albumin
BDNF: Brain-derived neurotrophic factor
AuNPs: Gold nanoparticles
MSNs: Mesoporous Silica nanoparticles
AchE: Acetylcholine esterase. malondialdehyde
MDA: Malondialdehyde
BACE-1: Beta-secretase 1
CBS: Cationic carbosilane
CeONPs: Cerium oxide nanoparticles
Lf: Lactoferrin
NSCs: Neural stem cells
SGZ: Sub granular zone
SVZ: Sub ventricular zone
VSV: Vesicular stomatitis virus
HSV: Herpes simplex virus
AAV: Adeno-associated virus
LV: lentiviral vectors
AV: Adenoviral vector
HIV-1: Human immunodeficiency virus
CED: Convection-enhanced delivery
ASOs: Antisense oligonucleotides
SL: Spliced leader
SMaRT: Spliceosome-mediated RNA transsplicing
siRNA: Small interfering RNA
RISC: RNA-induced silencing complex
Nrf2-HO-1: nuclear factor erythroid 2-related factor 2/heme oxygenase 1
NF-κB/COX-2: Nuclear factor-kappaB (NF-kappaB)/cyclooxygenase-2
iNOS: Inducible nitric oxide synthase
PDE10A: Phosphodiesterase 10 A
PPAR: Proliferator-activated receptor
